# Macrophage-state heterogeneity as the basis for host-directed therapy in tuberculosis

**DOI:** 10.3389/ftubr.2026.1969123

**Published:** 2026-10-28

**Authors:** Mohd Shariq, Nasreen Zafar Ehtesham, Seyed Ehtesham Hasnain

**Affiliations:** 1 Department of Life Sciences, GITAM School of Science, GITAM University, Hyderabad, Telangana, India; 2 Department of Life Sciences, Sharda School of Basic Sciences and Research, Sharda University, Greater Noida, Uttar Pradesh, India; 3 Department of Biochemical Engineering and Biotechnology, Indian Institute of Technology, Delhi Hauz Khas, New Delhi, India

**Keywords:** granuloma, host-directed therapy, immunometabolism, macrophage heterogeneity, regulated cell death, xenophagy

## Abstract

Tuberculosis (TB) persists partly because *Mycobacterium tuberculosis* (Mtb) occupies macrophages that differ in antimicrobial activity, nutrient use, inflammatory output, and drug exposure. This review examines how macrophage origin and lesion position influence phagosome maturation, responses to membrane damage, xenophagy, cytosolic sensing, regulated cell death, metabolism, and treatment response. Mtb can be taken up without lysosomal destruction, recognized by autophagy without completing lysosomal degradation, exposed to inflammatory signaling without effective killing, or retained in lesion regions with inadequate antibiotic concentrations. Evidence from single-cell studies, spatial analyses, experimental infection models, and clinical host-directed therapy studies supports evaluation of macrophage states rather than a fixed binary classification. Therapeutic candidates should be judged by linked host and bacterial outcomes, including completion of phagolysosomal or xenophagic degradation, viable bacterial burden, bacterial stress, preservation of macrophage and tissue function, lesion exposure, and compatibility with antimicrobial regimens. The evidence remains uneven: several mechanisms are well established, whereas many host-directed and nanomedicine approaches remain preclinical. Development should therefore use primary human macrophages, lesion-relevant models, lesion pharmacology, paired host-pathogen measurements, and separate microbiological, pathological, and functional endpoints. The aim is not broad immune stimulation but correction of defined macrophage and lesion states that permit persistence while preserving protective immunity.

## Graphical Abstract

**Figure:**
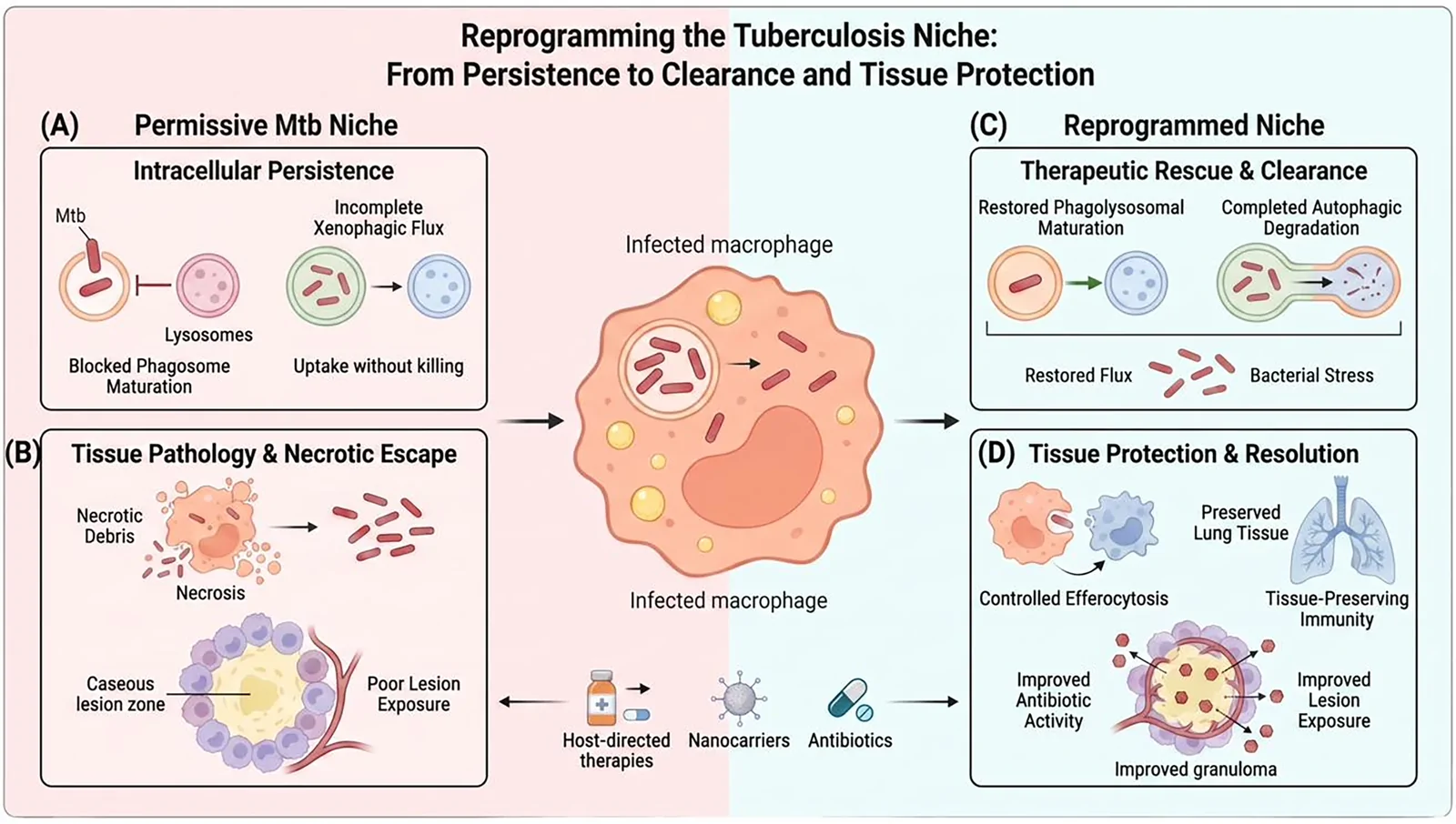
Reprogramming the Mtb-macrophage niche. (**A**) In the permissive *Mycobacterium tuberculosis* (Mtb) niche, intracellular persistence occurs when phagosome maturation is arrested and xenophagic recognition fails to progress to complete lysosomal degradation, leading to uptake without effective killing. (**B**) This permissiveness drives tissue pathology and necrotic escape, where caseous lesion zones and necrotic debris restrict local antibiotic exposure and promote bacterial survival and dissemination. (**C**) By applying state-matched host-directed therapies, targeted nanocarriers, and antibiotics, the infected macrophage can transition to a reprogrammed niche. This therapeutic rescue achieves bacterial clearance by restoring phagolysosomal maturation and driving completed autophagic degradation, thereby increasing bacterial stress. (**D**) This reprogrammed state ultimately promotes tissue protection and resolution by controlling efferocytosis, improving antibiotic activity and lesion exposure within the granuloma, and preserving functional lung tissue. Meaningful therapeutic benefit requires linked evidence of this restored host function, reduced viable Mtb, adequate lesion exposure, and overall tissue protection.

## 1 Introduction: tuberculosis persistence as a macrophage-state disease

Tuberculosis (TB) remains difficult to eliminate because *Mycobacterium tuberculosis* (Mtb) is a resilient pathogen that survives inside cells central to host defense. Macrophages ingest microbes, regulate inflammation, present antigen, and coordinate tissue responses, yet Mtb can alter receptor signaling, phagosome maturation, autophagy, metabolism, and cell death. The same cell can therefore act as an antimicrobial effector or as a protected bacterial niche. This host-pathogen relationship is a central determinant of TB persistence (1–7). The clinical burden of TB reflects more than bacterial drug resistance. Delayed diagnosis, human immunodeficiency virus (HIV) infection, diabetes, undernutrition, treatment toxicity, poor adherence, and post-TB lung damage all affect outcome. Within the lung, granulomas differ in oxygen tension, acidity (pH), lipid content, vascular access, inflammatory activity, and antimicrobial exposure. These conditions create distinct macrophage and bacterial states that are not represented by models that treat either population as uniform (8, 9).

Microbiological cure and tissue recovery should also be distinguished. Clinical host-directed therapy (HDT) studies have reported changes in lung function, inflammatory markers, or matrix destruction that do not always parallel early mycobacterial outcomes. For example, doxycycline reduced matrix metalloproteinase activity and cavity-associated tissue destruction without a clear early fall in mycobacterial load. Microbiological, pathological, and functional endpoints should therefore be defined separately (10–13).

The biological question addressed in this review is whether an infected macrophage can convert microbial recognition into degradative trafficking, bacterial stress, antibiotic susceptibility, and tissue-preserving immunity. Studies of macrophage heterogeneity show that origin, lesion location, receptor signaling, metabolic state, and phagolysosomal competence jointly influence restriction or persistence of Mtb (14, 15). This approach connects cell biology with granuloma pharmacology, HDT, and drug-delivery strategies. Accordingly, pathway activation is treated as an intermediate observation rather than a therapeutic endpoint. Evidence for a candidate intervention is stronger when changes in host pathways are linked to completed antimicrobial function, viable bacterial burden, lesion exposure, and a clinically relevant outcome (Table 1) (16, 17). These recurring macrophage-state defects, their functional measurements, pathological consequences, and corresponding therapeutic rationale are summarized in Table 1.

**Table 1:** Macrophage-state defects, functional measurements, and therapeutic rationale.

Failure mode	Mechanistic feature	Functional measurements	Pathological consequence	Therapeutic rationale	References
Uptake without maturation	Persistent early phagosome; impaired Rab7/LAMP1 acquisition and acidification	Rab5-to-Rab7 conversion; phagosomal pH; cathepsin activity; phagolysosomal fusion; intracellular CFU	Protected intracellular niche	Restore maturation without indiscriminate inflammation	(18, 19)
Damage recognition without flux	Galectin/ubiquitin/LC3 recruitment but incomplete lysosomal delivery	Galectin-8; TAX1BP1; p62 turnover; LC3 flux; bacterial localization	Autophagy-marker accumulation with bacterial persistence	Advance only candidates that complete degradative flux	(20–22)
Inflammation without killing	Type I IFN/IL-10 bias; receptor signaling that favors bacterial persistence; weak redox pressure	Cytokine ratios; ROS/NO; antigen presentation; intracellular CFU	Tissue injury with bacterial survival	Correct specific signaling defects rather than broadly stimulating immunity	(23–25)
Metabolic permissiveness	High OXPHOS with a reductive state and/or lipid loading	OCR/ECAR; NADH/NAD+; lipid droplets; bacterial redox reporters	Drug tolerance and foamy macrophages	Adjust metabolism while preserving mitochondrial viability	(26–28)
Necrotic escape	Ferroptosis, integrated stress response, and membrane failure	Lipid peroxidation; GPX4; cell-death mode; extracellular bacilli	Caseation, dissemination, and lung destruction	Prevent pathological necrosis and support safe efferocytosis	(29, 30)
Inadequate lesion exposure	Caseum binding, poor vascular access, and spatial sequestration	Lesion PK; mass-spectrometry imaging; unbound drug; caseum-binding assay	Persistent subpopulations and relapse	Design regimens and carriers around lesion-specific exposure	(31, 32)
Marker improvement without clinical benefit	Surrogate changes disconnected from bacterial or functional outcomes	CFU/culture; relapse; lung function; imaging; safety; PK/PD	Misleading treatment-development decisions	Use linked microbiological, pathological, and functional endpoints	(10, 11, 33)

CFU, colony-forming unit; ECAR, extracellular acidification rate; GPX4, glutathione peroxidase 4; IFN, interferon; IL, interleukin; LAMP1, lysosome-associated membrane protein 1; LC3, microtubule-associated protein 1 light chain 3; NADH/NAD+, reduced/oxidized nicotinamide adenine dinucleotide; NO, nitric oxide; OCR, oxygen consumption rate; OXPHOS, oxidative phosphorylation; p62, sequestosome 1; PK, pharmacokinetics; PK/PD, pharmacokinetics/pharmacodynamics; Rab, Ras-related protein family; ROS, reactive oxygen species; TAX1BP1, Tax1-binding protein 1.

This is a selective narrative review; we did not register a protocol or apply PRISMA methodology, consistent with the format expected of a Frontiers Review of this scope. The literature was assembled from PubMed, Web of Science, Scopus, and Google Scholar, and was updated through focused searches completed on 8 August 2026. We searched for records published from 1 January 2015 onward, combining TB or Mtb with macrophage or monocyte; phagosome maturation, Ras-related (Rab) proteins, lysosome, acidification, autophagy, xenophagy, galectins, ubiquitin, cytosolic deoxyribonucleic acid sensing, metabolism, redox control, lipids, mitochondria, cell death, efferocytosis, granuloma, caseum, lesion pharmacokinetics, HDT, and nanoparticle or inhaled delivery (representative Boolean string: (“Mycobacterium tuberculosis” OR Mtb) AND (macrophage* OR monocyte) AND (phagosome OR autophagy* OR xenophagy OR “cGAS-STING” OR cytokine* OR “cell death” OR immunometabolism* OR “trained immunity” OR granuloma* OR caseum OR “host-directed therapy” OR nanoparticle*); syntax was adapted to each database’s requirements). Within this window, studies published from 2020 onward were prioritized whenever they changed mechanistic or translational interpretation, because this period captures the single-cell, spatial-transcriptomic, and clinical host-directed therapy literature most relevant to the review’s translational focus. Foundational studies that established core paradigms used throughout this review (e.g., phagosome maturation arrest, xenophagy-mediated bacterial restriction, granuloma pharmacokinetics) were retained regardless of publication date via citation-tracking of key primary literature, because these original discovery papers remain the correct attribution for the concepts they defined and are not superseded by later work; where more recent studies extend or re-examine a classical finding in a current model system, both are cited together rather than the earlier paper being displaced; representative examples are noted in Table 1. Abstracts and titles were screened by the authors, with disagreements resolved by discussion; a record was retained if it reported primary experimental or clinical data, or a mechanistic synthesis, directly relevant to macrophage heterogeneity, Mtb trafficking or persistence, host-directed therapy, or lesion-targeted delivery, with review articles retained mainly for context and primary studies preferred for specific mechanistic or clinical claims. Because this was not a systematic review, formal eligibility criteria, language filters, and a risk-of-bias instrument were not prespecified, and no meta-analysis was performed; we mitigate the resulting risk of selective emphasis by reporting the evidence level explicitly for every major claim (Tables 1–3) and by citing existing systematic reviews and meta-analyses where available for specific sub-questions (e.g., anti-inflammatory adjuncts) (34, 35). When reports conflicted, greater weight was given to evidence from primary human cells, lesion-relevant systems, paired host-pathogen measurements, and studies that directly measured bacterial viability or clinical outcomes.

## 2 Macrophage ontogeny, heterogeneity, and spatially defined niches

The first question is how cell origin and location shape the intracellular environment encountered by Mtb. The infected lung contains embryonically seeded, self-renewing resident alveolar macrophages and recruited monocytes that can become interstitial or monocyte-derived macrophages (Figure 1A). Foamy, epithelioid, multinucleated, inflammatory, and resolution-associated states emerge within lesions (Figure 1B). These populations differ in epigenetic state, nutrient use, receptor expression, and anatomical position. The traditional classically activated (M1) and alternatively activated (M2) classification is therefore inadequate for TB; origin, location, metabolic program, phagolysosomal competence, bacterial stress, and lymphocyte interaction provide a more useful description (14, 15).

**Figure 1:**
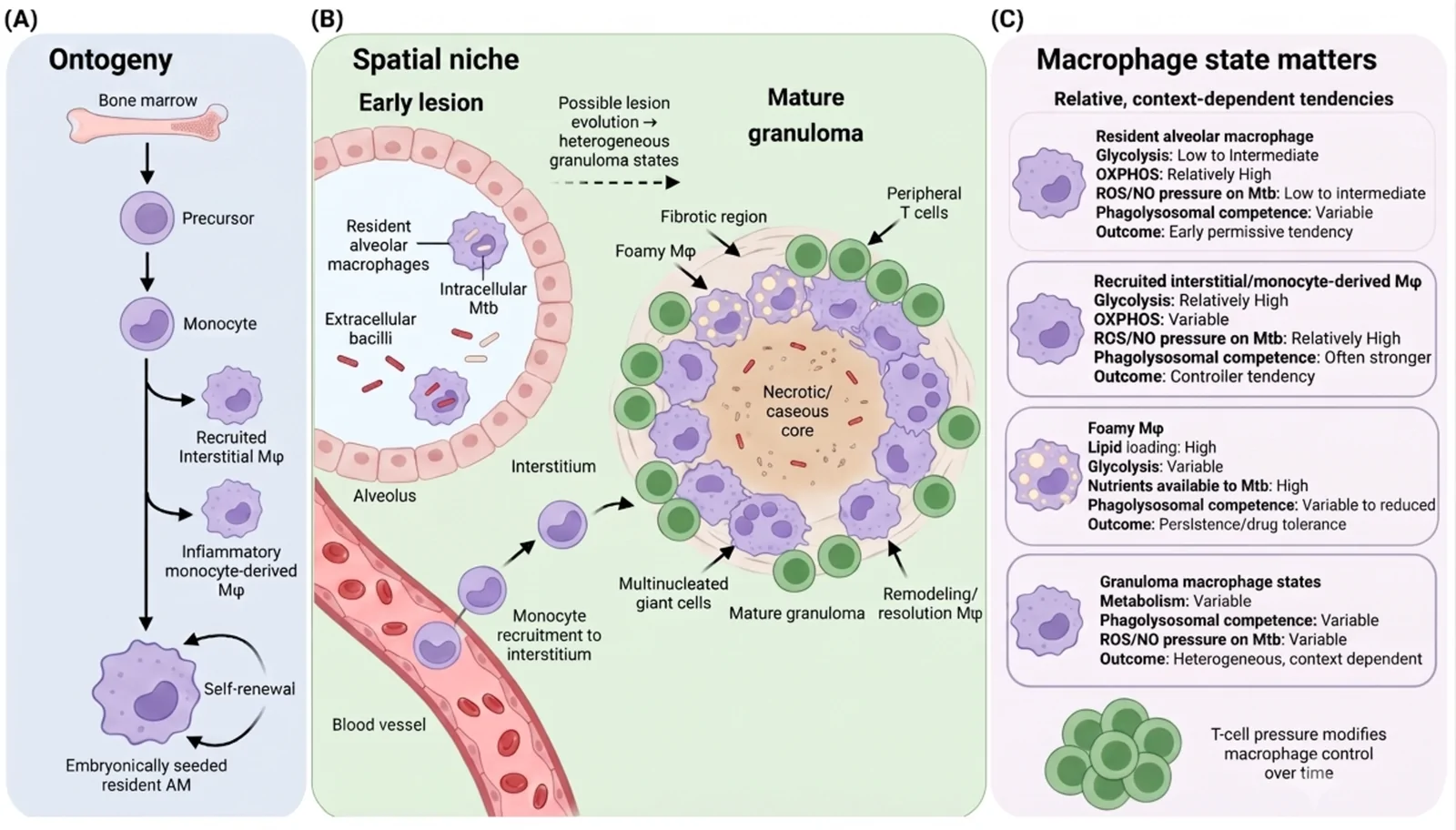
Macrophage origin, lesion position, and functional state shape the Mtb niche. **(A)** Mycobacterium tuberculosis (Mtb) encounters resident alveolar macrophages and blood-derived monocyte populations with different origins and developmental trajectories, including self-renewing resident cells and recruited interstitial or inflammatory monocyte-derived macrophages. **(B)** As early lesions evolve into mature granulomas, macrophages occupy distinct anatomical and metabolic spatial niches, ranging from the vascularized interstitium to fibrotic regions, foamy niches, and necrotic or caseous cores. **(C)** The relative, context-dependent tendencies of these macrophage states, established predominantly in mouse infection models, influence glycolysis, oxidative phosphorylation (OXPHOS), ROS/NO pressure, and phagolysosomal competence, and are associated with a more permissive (e.g., early resident or foamy macrophages) or restrictive (e.g., NOS2-high recruited macrophages, in mice) phenotype in that setting. These states are not fixed, can be modified by T-cell pressure over time, and should not be assumed to map directly onto human macrophage populations without direct comparison.

In mouse aerosol-infection models, resident alveolar macrophages are an important early niche: their surfactant-handling and oxidative programs can favor bacterial growth, whereas recruited interstitial and monocyte-derived macrophages often impose stronger glycolytic and nitric oxide-associated stress (Figure 1C) (15, 36–40). These are relative tendencies observed principally in murine infection, not fixed cross-species properties, and bacterial control varies within each population and changes as adaptive immunity develops. A single-cell RNA-sequencing study of the infected mouse lung shows heterogeneity within the same lineage: macrophages positive for cluster of differentiation 38 (CD38) display epigenetic priming and stronger restriction of Mtb, while other alveolar macrophages support early growth; intranasal Bacille Calmette-Guérin (BCG) vaccination increased macrophage populations with stronger bacterial control in this mouse model, raising the possibility that mucosal vaccination can alter the cellular niche before exposure (41). Whether the same CD38-associated priming and BCG-induced shift occur in human alveolar macrophages has not yet been established.

Genome-wide perturbation can identify host regulators that are missed by bulk measurements. A genome-wide clustered regularly interspaced short palindromic repeats (CRISPR) screen in Mtb-infected mouse macrophages showed that disruption of the glucose-induced degradation/C-terminal to Lis homology (GID/CTLH) ubiquitin ligase complex increased adenosine monophosphate-activated protein kinase (AMPK) signaling, glycolysis, autophagic flux, resistance to necrosis, and bacterial stress (42). This experimental result, from a mouse macrophage system, shows why candidate targets are more informative when a host change is linked to the condition of intracellular Mtb; confirmation in primary human macrophages would strengthen its translational relevance.

Location within the lesion adds another source of variation. Granulomas within one host can differ in macrophage, lymphocyte, neutrophil, and fibroblast content and in bacterial replication. Spatial transcriptomic studies in mouse (43, 44) and zebrafish (45) granuloma models have identified organized transcriptional layers, with osteopontin (SPP1)-positive macrophages forming the dominant population and acting as a trajectory hub for macrophage polarization; in a *Mycobacterium marinum-*zebrafish model, spp1 was upregulated in infected macrophages, and genetic ablation of spp1 impaired granuloma formation and reduced host survival, directly linking this macrophage program to granuloma-level host defense (45). A separate spatial comparison of human pulmonary and omental Mtb granulomas, using microbiologically confirmed clinical biopsy tissue, found a more lymphocyte/B-cell- and fibroblast-rich composition in the lung versus a myeloid- and fibroblast-dominated composition in the omentum, with a shared collagen/fibrotic signature at both sites; a human induced pluripotent stem cell (iPSC)-derived lung organoid-macrophage co-culture model further showed that thrombospondin-1 (THBS1) reduced extracellular-matrix-remodeling protein expression, a potential host-directed lead (46). The osteopontin/SPP1-driven trajectory and layered architecture described above were established in animal models (43–45); the compartment-specific immune composition and fibrotic signature were observed directly in human tissue (46), and the extent to which the animal-model findings generalize to human granulomas remains to be tested. These spatial differences are biologically important because the same cytokine or metabolic program can have different effects at the airway surface, cellular granuloma rim, or necrotic border. T-cell-derived signals can increase antimicrobial pressure as adaptive immunity develops, while fibroblast-rich or lipid-rich regions can restrict cell movement and alter nutrient availability. Foamy macrophages may arise from several precursor populations and should be described by their lipid-loaded state rather than treated as a separate fixed lineage. Similarly, multinucleated giant cells and resolution-associated macrophages are better viewed as lesion-associated states whose functions depend on location and time. This distinction matters for therapy: a target enriched in a macrophage population that permits persistence is useful only if the intervention reaches that population and preserves functions required for local containment (15, 41).

Macrophage states also change over time, including shifts from early glycolytic activity toward lipid use, oxidative phosphorylation, or immune regulation. Nuclear receptor signaling links inflammatory activity with nutrient handling (27, 47, 48). For treatment, the relevant target is therefore a defined macrophage state in a defined lesion compartment and disease stage, rather than an average lung macrophage (Figures 1A–C).

## 3 Phagolysosomal competence: how Mtb uncouples uptake from lysosomal killing

The key trafficking question is whether bacterial uptake is followed by lysosomal delivery. Early Mtb-containing phagosomes acquire Rab5 and phosphatidylinositol 3-phosphate, then normally gain Rab7, lysosome-associated membrane proteins (LAMPs), acidity, and lysosomal fusion (Figure 2A). Virulent Mtb can delay this progression and preserve a less degradative compartment (Figure 2B) (18, 19, 49–51). H37Rv can also block autophagic progression more efficiently than H37Ra, showing that association with microtubule-associated protein 1 light chain 3 (LC3) does not by itself establish bacterial destruction (52–55). Rab7 recruitment links phagosome maturation with later autophagic trafficking. H37Ra-containing LC3-positive compartments can acquire Rab7 and progress toward lysosomal fusion, whereas H37Rv limits this step; the PhoP response regulator and early secreted antigenic target of 6 kilodaltons (ESAT-6) have been implicated in the block (52). Regulatory differences between H37Rv and H37Ra may also extend beyond coding sequence, as comparative chromosome-contact studies identified distinct genome organization in the two strains (56).

**Figure 2:**
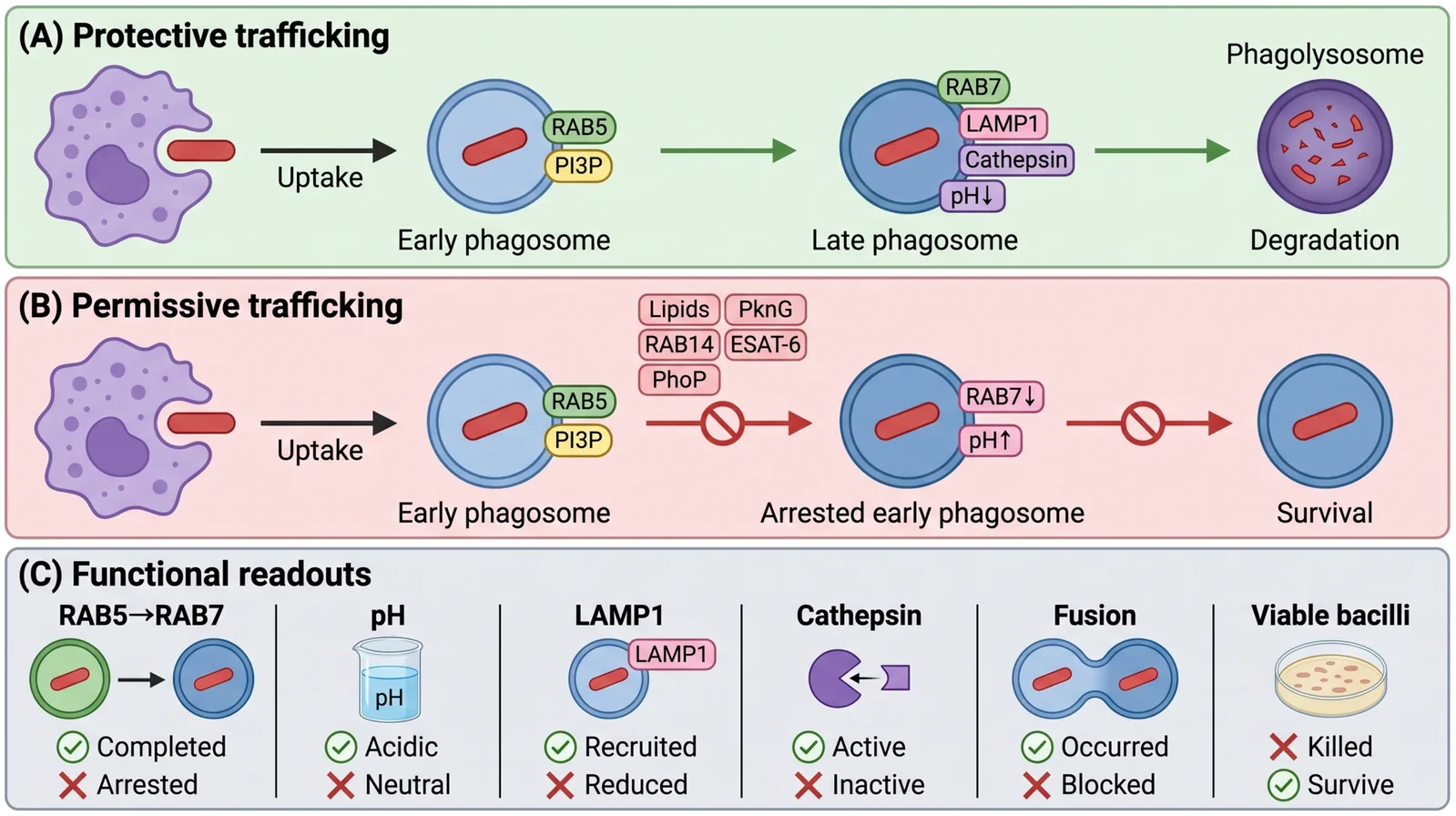
Phagosomal trafficking separates bacterial uptake from lysosomal destruction. **(A)** Protective trafficking proceeds from a Rab5 and PI3P-positive early phagosome toward Rab7 acquisition, acidification (low pH), lysosome-associated membrane protein 1 (LAMP1) recruitment, cathepsin activity, and lysosomal fusion, culminating in bacterial degradation. **(B)** Permissive trafficking occurs when virulent Mtb utilizes factors like mycobacterial lipids, PknG, Rab14, ESAT-6, and PhoP to interrupt this sequence. This blocks Rab7 recruitment and prevents acidification, causing maturation arrest and promoting bacterial survival. **(C)** Key functional readouts that distinguish completed protective maturation from arrested permissive trafficking include Rab5-to-Rab7 progression, compartment pH levels, LAMP1 recruitment, cathepsin activity, fusion events, and the presence of viable bacilli.

Host and bacterial heterogeneity both influence acidification. Earlier studies established strong differences among macrophage sources, while a 2026 single-cell study showed that clinical Mtb lineage and macrophage phenotype interact to determine phagosomal acidification (57). This new evidence supports direct measurement of trafficking in the relevant host cell and strain, but it does not justify a simple restrictive-versus-permissive ranking based on lineage or drug-resistance category alone (1, 2, 58). The maturation defect is also graded rather than all-or-none. Individual Mtb-containing compartments can differ in proton-pump acquisition, membrane composition, Rab conversion, and access to lysosomal enzymes within the same infected cell. These differences help explain why measurements based only on average fluorescence or total cellular lysosome content can miss persistent bacterial subpopulations. When comparing strains or macrophage sources, experiments should therefore report single-cell or single-phagosome distributions where possible and should pair compartmental measurements with viable bacterial counts. This approach is especially important for HDT candidates that are expected to restore trafficking, because a modest shift in the population average may coexist with a persistent fraction of poorly acidified, drug-tolerant bacilli (57–59).

For therapeutic studies, useful measurements include Rab5-to-Rab7 progression, acquisition of lysosome-associated membrane protein 1 (LAMP1), compartment pH, cathepsin activity, phagosome-lysosome fusion, and viable intracellular bacilli (Figure 2C). These measurements distinguish successful maturation from increased uptake or accumulation of an early trafficking marker (60–62) (Figures 2A–C).

## 4 Cytosolic-sensing states: xenophagy and incomplete autophagic degradation

When phagosome maturation is disrupted, damaged Mtb-containing compartments can enter selective autophagy pathways. Galectins, ubiquitin, LC3-family proteins, and cargo adaptors can mark damaged membranes or exposed bacilli for lysosomal delivery (Figure 3A) (20, 21, 60, 63). The key question is whether recognition progresses to a degradative compartment, because LC3 or sequestosome 1 (p62) can accumulate while viable bacilli persist (Figure 3B) (22, 64, 65). ESAT-6 secretion system 1 (ESX-1)-dependent phagosomal permeabilization exposes Mtb deoxyribonucleic acid (DNA) to cytosolic cyclic GMP-AMP synthase (cGAS). cGAS produces cyclic GMP-AMP (cGAMP), which activates stimulator of interferon genes (STING; gene STING1), TANK-binding kinase 1 (TBK1), and interferon regulatory factor 3 (IRF3) (Figure 4D) (66–69). This pathway can support selective autophagic targeting, but it can also drive type I interferon programs associated with susceptibility. Its effect depends on signal timing, strength, cell type, and the surrounding cytokine environment (24, 70–72).

**Figure 3:**
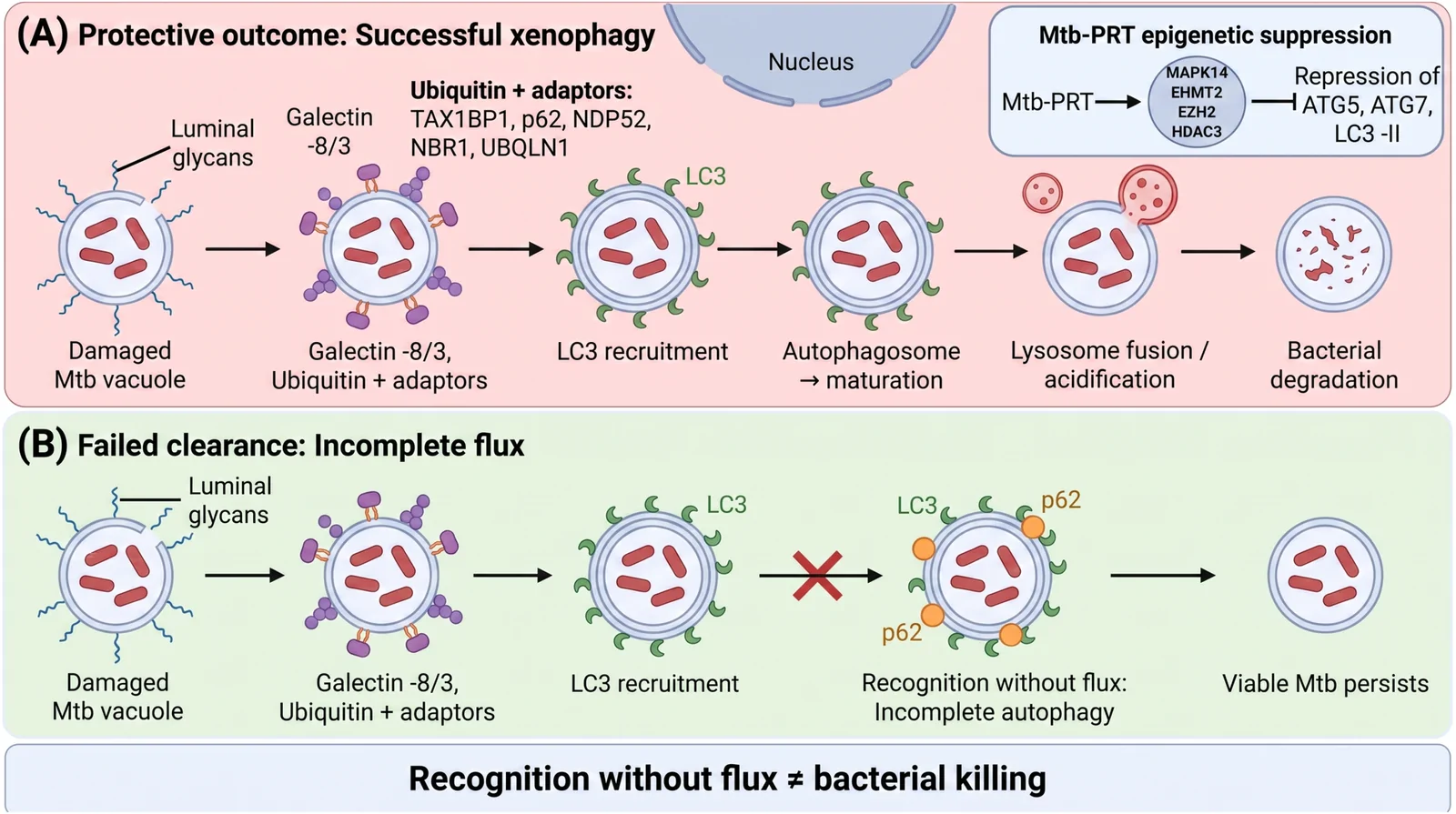
Recognition of vacuolar damage does not necessarily result in bacterial degradation. **(A)** In a protective outcome demonstrating successful xenophagy, damaged Mtb-containing vacuoles expose luminal glycans that recruit galectins (-8/3), ubiquitin, and selective-autophagy adaptors (TAX1BP1, p62, NDP52, NBR1, UBQLN1), followed by LC3 recruitment. This progresses to autophagosome maturation, lysosomal fusion, and bacterial degradation. Mtb phosphoribosyltransferase (Mtb-PRT) can antagonize this protective pathway by epigenetically suppressing autophagy-related gene expression (repressing ATG5, ATG7, LC3-II via MAPK14, EHMT2, EZH2, and HDAC3). **(B)** Failed clearance occurs when incomplete flux leaves viable Mtb intact, demonstrating that early recognition steps (LC3 and p62 accumulation) without successful progression to lysosomal degradation do not equate to bacterial killing.

**Figure 4:**
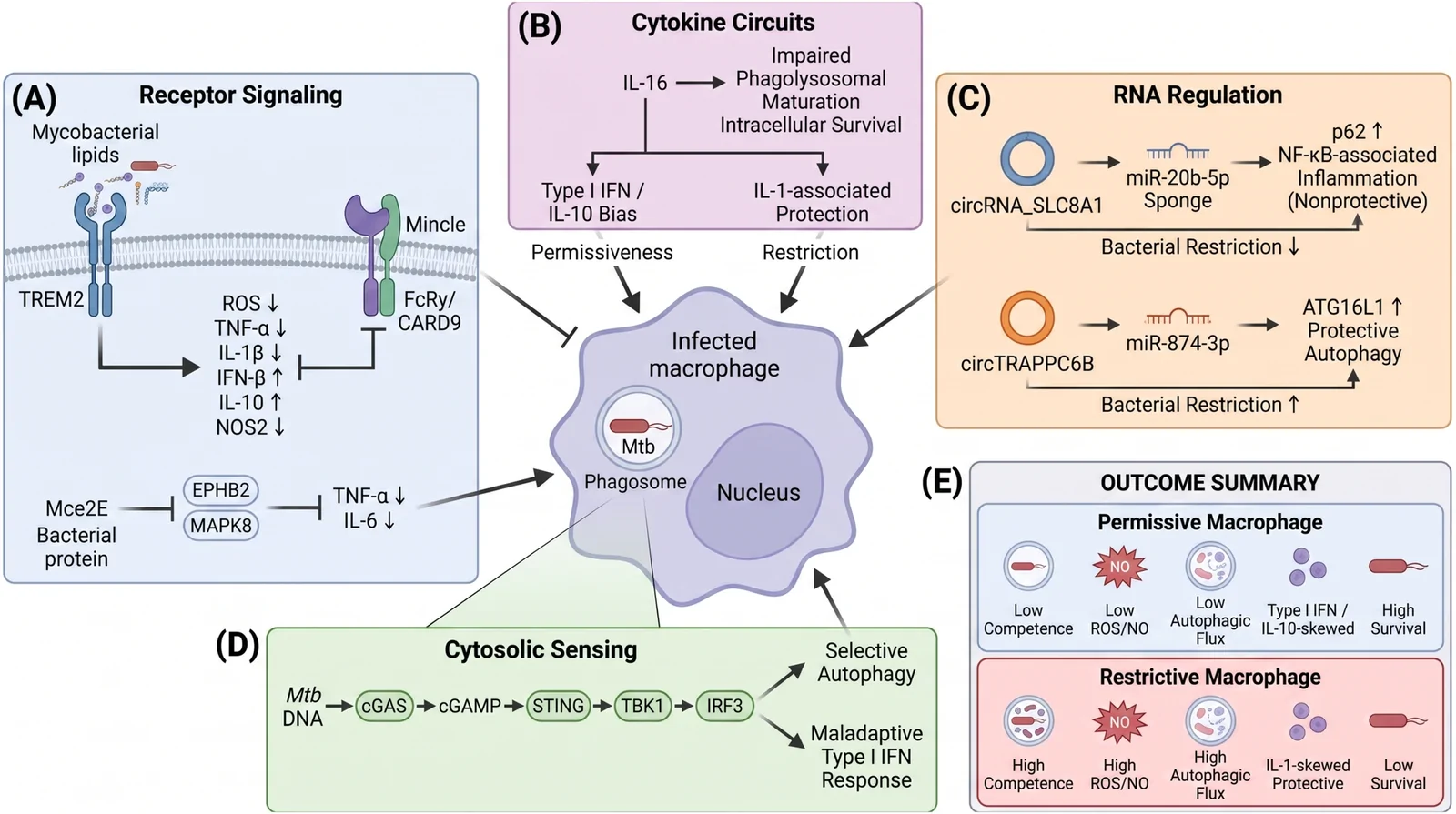
Receptor, cytokine, cytosolic-sensing, and ribonucleic acid pathways influence bacterial control. **(A)** Receptor signaling cascades on the infected macrophage, such as TREM2 binding mycobacterial lipids or Mce2E inhibiting EPHB2/MAPK8, can dampen antimicrobial pressure (lowering ROS, TNF-alpha, IL-1beta) to favor persistence, whereas Mincle/FcR-gamma signaling promotes restriction. **(B)** Cytokine circuits demonstrate that IL-16 impairs phagolysosomal maturation, and a type I interferon or IL-10 bias supports permissiveness, whereas IL-1-associated responses offer protection. **(C)** RNA regulation involves distinct molecular circuits, such as circRNA_SLC8A1 sponging miR-20b-5p to increase nonprotective p62 and NF-kappaB inflammation, versus circTRAPPC6B sponging miR-874-3p to increase protective ATG16L1-mediated autophagy. **(D)** Cytosolic sensing of Mtb DNA via cGAS-STING-TBK1-IRF3 can drive either selective autophagy or a maladaptive type I interferon response. **(E)** The outcome summary contrasts permissive and restrictive macrophage states based on functional competence, ROS/NO production, autophagic flux, cytokine skewing, and ultimate bacterial survival.

Recent experimental evidence adds an important qualification. In human macrophages, Ca2+ leakage from damaged Mtb phagosomes can trigger vacuolar-type adenosine triphosphatase (V-ATPase)- and autophagy-related 16-like 1 (ATG16L1)-dependent LC3 lipidation on multiple membranes without canonical xenophagic delivery to lysosomes; this response nevertheless limits membrane damage and bacterial replication (73). Thus, LC3 recruitment can reflect different forms of membrane stress control and must be interpreted with compartmental and functional measurements. Ubiquitin-dependent recognition recruits adaptors including p62, calcium-binding and coiled-coil domain-containing protein 2 (CALCOCO2, also called NDP52), NBR1 autophagy cargo receptor (NBR1), and related proteins that connect ubiquitinated cargo with LC3-family proteins. Ubiquilin-1 can promote accumulation of ubiquitin, p62, and LC3 around Mtb after phagosomal damage in interferon-gamma (IFN-gamma)-activated macrophages (65, 74, 75).

Mtb can also suppress autophagy at the level of host gene expression. Its phosphoribosyltransferase (Mtb-PRT) induces repressive chromatin changes involving mitogen-activated protein kinase 14 (MAPK14), euchromatic histone lysine methyltransferase 2 (EHMT2), enhancer of zeste homolog 2 (EZH2), and histone deacetylase 3 (HDAC3) at autophagy-related loci. These changes reduce autophagy-related 5 (ATG5), autophagy-related 7 (ATG7), and lipidated LC3 (LC3-II), while increasing p62 and bacterial survival (Figure 3A) (76). This is mechanistic evidence from experimental models rather than clinical proof of benefit from targeting the pathway. Animal studies support a protective role for autophagy during acute infection but also show that ATG proteins have non-autophagic functions and that outcomes depend on deletion efficiency, infection dose, and cell type. More complete depletion of ATG7 or ATG16L1 increased Mtb growth, while other models linked autophagy to control of harmful myeloid responses and maintenance of T-cell immunity (77–80).

The apparent disagreement among autophagy studies also reflects the assay used. Static LC3 puncta, p62 abundance, or ATG expression cannot distinguish increased autophagosome formation from impaired clearance without additional flux measurements. Genetic deletion can also alter inflammation, antigen presentation, or cell survival independently of canonical xenophagy. For this reason, inhibitor controls, time-resolved imaging, lysosomal colocalization, and bacterial viability are needed to assign a change to completed degradative flux. The recent Ca2+-leakage work further shows that LC3 lipidation may limit membrane damage without delivering bacilli to lysosomes, reinforcing the need to identify the membrane compartment and the functional consequence before describing a response as xenophagy (73, 77).

Human genetic associations with autophagy-related genes remain heterogeneous and do not yet support genotype-based selection of autophagy-directed HDT (77, 81). For treatment development, interventions should be tested for delivery of Mtb to a degradative compartment and for reduction of viable bacilli rather than selected from autophagy markers alone.

## 5 Endolysosomal quality-control states: lysosomal-damage sensing

Lysosomal quality control asks whether membrane injury is detected, repaired, or routed to degradation before the compartment becomes a source of cytosolic escape. Damage exposes luminal glycans to cytosolic galectins. Galectin-8 can recruit Tax1-binding protein 1 (TAX1BP1) and related selective-autophagy proteins, while the balance of galectin-3 and galectin-8 depends partly on membrane glycan composition (61, 62). Mtb interferes with this network at several points. Protein kinase G can activate early autophagy-associated signals while maintaining maturation arrest through Rab14 and related regulators (82). Mycobacterial lipids also change lysosome abundance and positioning, so an increase in lysosome number does not necessarily mean that bacilli enter bactericidal compartments (59).

Transcription factor EB (TFEB) links nutrient sensing with lysosomal biogenesis, but its effect depends on the setting. Infection, starvation, mechanistic target of rapamycin complex 1 (mTORC1), calcineurin, lipids, and cellular stress can produce different outcomes. Therapeutic TFEB activation is most convincing when it restores acidity, protease activity, cargo delivery, and bacterial killing in primary human macrophages (83). Membrane repair and organelle replacement should also be considered separately. A damaged phagosome may be repaired, removed by selective autophagy, or progress to wider endolysosomal failure. Each outcome has different consequences for cytosolic antigen exposure, bacterial escape, inflammatory signaling, and cell death. A treatment that increases lysosome biogenesis could be useful when degradative capacity is limiting, yet insufficient if Mtb-containing compartments remain physically excluded from those lysosomes. Conversely, forcing membrane damage to expose bacterial material may increase cytosolic sensing but also increase lytic death. These trade-offs argue for experiments that combine membrane-integrity reporters with trafficking and bacterial-survival measurements instead of assuming that more damage sensing or more lysosomes is beneficial (59, 84). The evidence therefore supports a sequence of measurements rather than reliance on one marker: phagosomal pH, Rab7 and LAMP1 acquisition, cathepsin activity, galectin and ubiquitin dynamics, LC3 turnover with and without lysosomal inhibition, bacterial localization, and viable intracellular bacilli (Figure 2C). Cell death and inflammatory output should be measured in parallel when lysosomal pathways are manipulated (81, 85).

This interpretation is also consistent with evidence that Mtb can alter trafficking beyond the bacillus-containing compartment, and that ESAT-6-mediated membrane injury has different consequences depending on acidification, repair, selective autophagy, and lytic cell death (84, 86). The therapeutic goal is restoration of endolysosomal competence without increasing destructive membrane injury (Figures 3A,B).

## 6 Signaling-defined states: receptor, cytokine, and ribonucleic acid control of macrophage function

The next question is how extracellular and cytosolic signals affect bacterial control. Mtb changes macrophage state through receptor choice, bacterial effectors, cytokines, and ribonucleic acid (RNA)-mediated regulation (87–91). Claims about mechanism are strongest when a bacterial or host factor is directly linked to a change in antimicrobial function; structural or expression data alone are not sufficient. Rv2231c provides one experimentally supported example of an Mtb protein that suppresses host defense (92, 93). At the cell surface, entry receptors influence signaling as well as uptake. Triggering receptor expressed on myeloid cells 2 (TREM2) recognizes non-glycosylated mycolic acid-containing lipids and can oppose signaling through macrophage-inducible C-type lectin (Mincle), the Fc receptor gamma chain (FcR-gamma), and caspase recruitment domain-containing protein 9 (CARD9). In human macrophages, TREM2-mediated uptake reduces tumor necrosis factor-alpha (TNF-alpha), interleukin-1beta (IL-1beta), and reactive oxygen species (ROS), while increasing interferon-beta (IFN-beta) and interleukin-10 (IL-10); genetic deletion or antibody blockade restored ROS-associated restriction in experimental systems (Figure 4A) (23, 94, 95). This is mechanistic evidence, not yet a clinical basis for TREM2-directed therapy.

The Mtb mammalian cell entry protein Mce2E suppresses macrophage innate responses by inhibiting EPH receptor B2 (EPHB2) and mitogen-activated protein kinase 8 (MAPK8), with reduced TNF-alpha and interleukin-6 (IL-6) expression (96). Its effects differ in epithelial cells, illustrating that an Mtb effector can have host-cell-specific actions (97–99). Host cytokines can reinforce the same macrophage state. Interleukin-16 (IL-16) is increased in active TB and, in zebrafish and mouse models, promotes intracellular growth by impairing phagolysosome maturation and altering nuclear receptor subfamily 1 group D member 1 (NR1D1)-linked control of IL-10. Reducing IL-16 improved pathology or survival in these preclinical models (Figure 4B) (100).

RNA regulation provides an additional level of control. A circular RNA derived from SLC8A1 (circRNA_SLC8A1) is increased in patients with TB and in infected macrophages; experimental work links it to microRNA-20b-5p (miR-20b-5p) sequestration, increased p62, nuclear factor kappa B (NF-kappaB) activation, altered ROS and apoptosis, and greater intracellular Mtb survival (Figure 4C) (101). The human association does not by itself establish therapeutic efficacy, and p62 accumulation should not be interpreted as completed xenophagy. A circular RNA derived from TRAPPC6B (circTRAPPC6B) has been linked to microRNA-874-3p (miR-874-3p)- and ATG16L1-dependent autophagy and bacterial restriction (Figure 4C). A later galactose-targeted microparticle study combined this RNA with rifampicin in macrophage and mouse models (102, 103). These are preclinical findings. They support study of this specific RNA pathway rather than a general conclusion that noncoding-RNA manipulation will improve bacterial clearance. Cytokine pathways should be interpreted with the same caution. Type I interferon and IL-10 can oppose interleukin-1 (IL-1)-associated protection in some models, but their effects vary with infection stage, cell type, and signal intensity (104–106). TNF-alpha is required for important antimicrobial and granuloma functions, yet excessive TNF-alpha can contribute to tissue injury. Thus, receptor or cytokine blockade is unlikely to have a single effect across all lesions. The most informative studies will define the starting macrophage state, measure the intended signaling change, and determine whether bacterial control improves without unacceptable loss of cell viability or tissue containment (24, 25, 71, 72).

A single-cell RNA-sequencing study of the infected mouse lung places these pathways within a mixed macrophage population: interstitial macrophages positive for inducible nitric oxide synthase (NOS2) impose high bacterial stress in this model, whereas other states show lower stress or programs associated with repair (Figure 4E) (41). NOS2-dependent restriction is a well-established antimicrobial mechanism in mouse macrophages, but its expression and functional importance are more limited and variable in human macrophages (14, 15); this NOS2-based classification should therefore not be extrapolated to human disease without direct testing. Accordingly, receptor, cytokine, or RNA interventions should be matched to the infected macrophage state and the species/model system in which it was defined and tested against bacterial as well as host outcomes (36–39, 58, 68) (Figures 4A–E).

## 7 Macrophage fate states: regulated cell death, efferocytosis, and escape from containment

The biological question for regulated cell death is whether an infected cell contains bacilli, transfers them safely to another phagocyte, or releases them into tissue. Apoptosis can package bacilli and antigens in membrane-bound bodies and support efferocytic uptake (Figure 5A), but its benefit depends on bacterial burden, timing, and the degradative capacity of recipient cells. Necrotic membrane failure more readily releases viable bacteria and inflammatory contents (85, 107–110). Ferroptosis links iron-dependent lipid peroxidation with necrotic tissue injury (Figure 5D). In experimental TB, ferrostatin-1 reduced ferroptotic death and improved bacterial control and pathology (29). This is preclinical evidence. Any therapeutic strategy would need to show that limiting ferroptosis preserves tissue without maintaining heavily infected cells that should be cleared.

**Figure 5:**
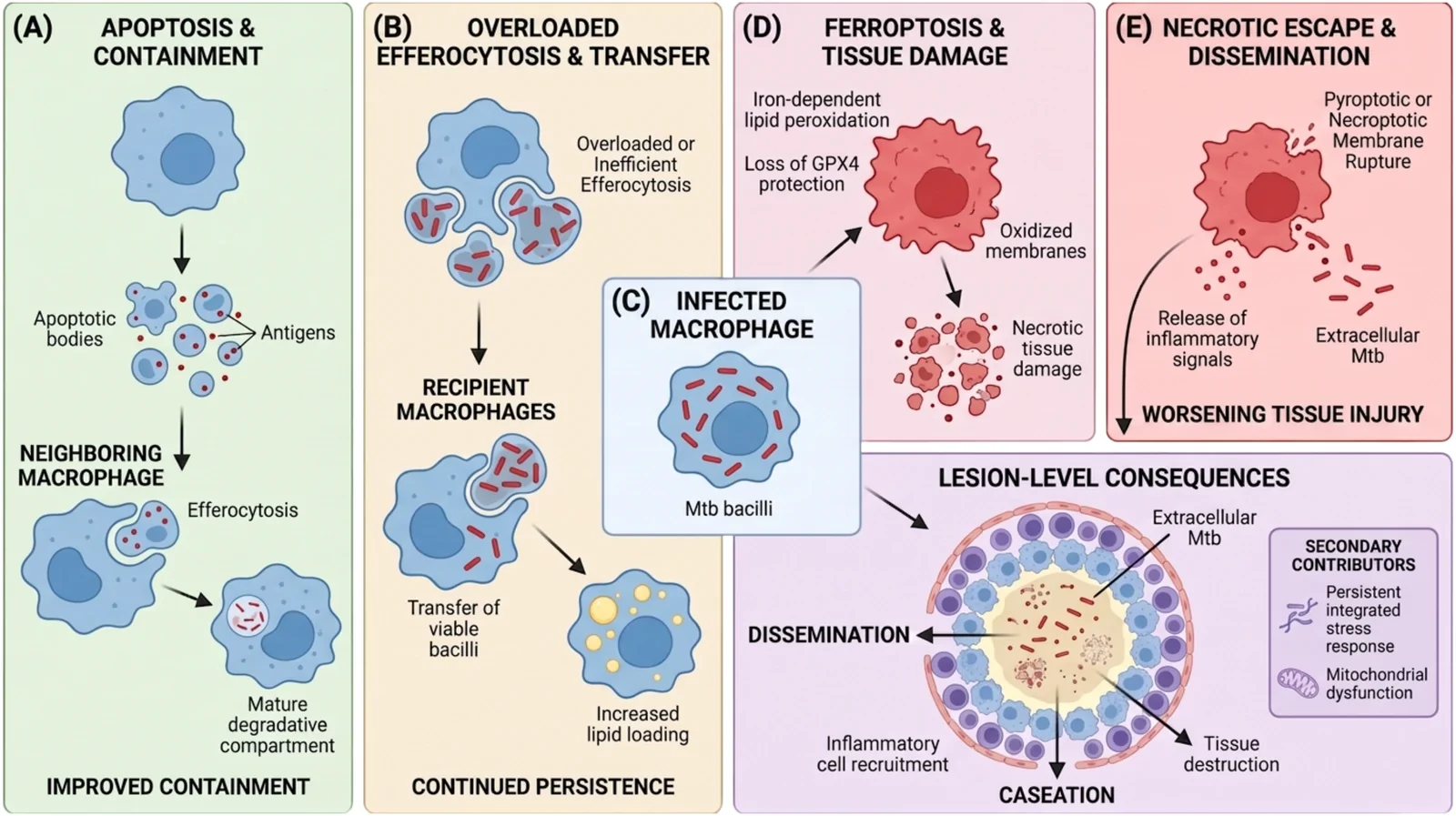
The fate of an infected macrophage can lead to containment, bacterial transfer, or extracellular escape. **(A)** Apoptosis followed by effective efferocytosis by neighboring macrophages can support successful antigen transfer and delivery of Mtb to a mature degradative compartment, improving containment. **(B)** Overloaded or inefficient efferocytosis can result in the transfer of viable bacilli to recipient macrophages, leading to increased lipid loading and continued persistence. **(C)** Central infected macrophages supply bacilli and inflammatory signals to the lesion. **(D)** Ferroptosis, driven by iron-dependent lipid peroxidation and loss of GPX4 protection, causes necrotic tissue damage. **(E)** Necrotic escape through pyroptotic or necroptotic membrane rupture releases inflammatory signals and extracellular Mtb, worsening tissue injury. Lesion-level consequences of these failure modes include dissemination, inflammatory cell recruitment, caseation, and tissue destruction, exacerbated by persistent integrated stress responses and mitochondrial dysfunction.

Persistent integrated stress responses can also promote mitochondrial dysfunction, type I interferon signaling, and granuloma necrosis in susceptible hosts (30). Because short stress responses are required for adaptation, the therapeutic target is sustained pathological stress rather than stress signaling in general. Efferocytosis is similarly conditional. Uptake of apoptotic infected cells may deliver bacilli to a more mature degradative compartment, but repeated or inefficient efferocytosis can transfer viable bacilli, overload recipient macrophages, and increase lipid accumulation (Figure 5B). Experiments should therefore follow the fate of bacteria after transfer, not only the uptake of apoptotic material.

A useful experimental approach is to follow four linked compartments: the primary infected macrophage, apoptotic or necrotic material released from it, the recipient phagocyte, and extracellular bacilli. This separates true bacterial killing from simple transfer between cells. It also allows measurement of whether repeated efferocytosis increases lipid loading or exhausts lysosomal capacity in recipient macrophages. At lesion scale, the same distinction determines whether cell death supports resolution or promotes caseation. Candidate cell-death HDTs should therefore report bacterial location, viable burden, inflammatory mediator release, and tissue injury together, because improvement in one measurement can be accompanied by worsening of another.

Pyroptosis and necroptosis can release inflammatory mediators and may either alert neighboring cells or worsen tissue injury (Figure 5E). Because pharmacological inhibitors can have off-target and species-specific effects, genetic and biochemical confirmation in human macrophages is important (111). The therapeutic aim is controlled containment and tissue preservation, not promotion of a single death pathway in every infected macrophage (Figures 5A–E).

## 8 Immunometabolism, redox heterogeneity, and antibiotic tolerance

The metabolic question is how the host cell changes the physiological state and drug response of intracellular Mtb. Macrophage glycolysis, oxidative phosphorylation, lipid handling, redox buffering, iron metabolism, and mitochondrial integrity influence bacterial growth, stress, and cell survival (85). Mtb infection can increase glycolysis, lactate production, pentose-phosphate pathway activity, and tricarboxylic-acid-cycle remodeling (17). Metabolomic studies also identify changes in itaconate, redox metabolites, nucleotide sugars, and cholesterol metabolism (47, 112). These patterns vary by cell state and should not be assigned as fixed metabolic signatures of a bacterial lineage or resistance phenotype.

Mitochondrial integrity is part of this antimicrobial balance. Mtb reduces sirtuin 3 (SIRT3) in macrophages, with disruption of mitochondrial pathways, increased oxidative stress, and cell death; SIRT3-deficient mice have higher bacterial burdens and more severe pathology (28, 113). mTORC1 also has setting-dependent functions: although inhibition of mechanistic target of rapamycin (mTOR) can induce autophagy, mTORC1 can preserve mitochondrial energy production during ESAT-6-associated stress (114). Lipid-rich macrophages provide another persistence niche. Mtb can use host cholesterol and fatty acids, while infection promotes cholesterol uptake and esterification, lipid-droplet formation, and impaired efflux (115, 116). Foamy macrophages and lipid-rich lesion material can support persistence, caseation, and drug tolerance, although lipid droplets may have protective functions in other settings (26, 117–123).

Host and bacterial metabolism can be measured together. Cholestenone, produced through Mtb modification of host cholesterol, correlates with pathogen burden and distinguished TB from symptomatic non-TB controls in separate clinical cohorts (112). Such metabolites are candidate biomarkers, but their value for treatment selection or relapse prediction requires independent validation. Direct bacterial redox reporters provide a clearer connection between host metabolism and bacterial response. In a 2025 preclinical study, macrophages with high oxidative phosphorylation and low glycolysis contained more reductive, drug-tolerant Mtb (Figure 6B), whereas glycolytically active macrophages generated mitochondrial ROS that increased bacterial oxidative stress and antibiotic activity (Figure 6A). Nuclear factor erythroid 2-related factor 2 (NRF2; gene NFE2L2) contributed to the reductive niche (124).

**Figure 6:**
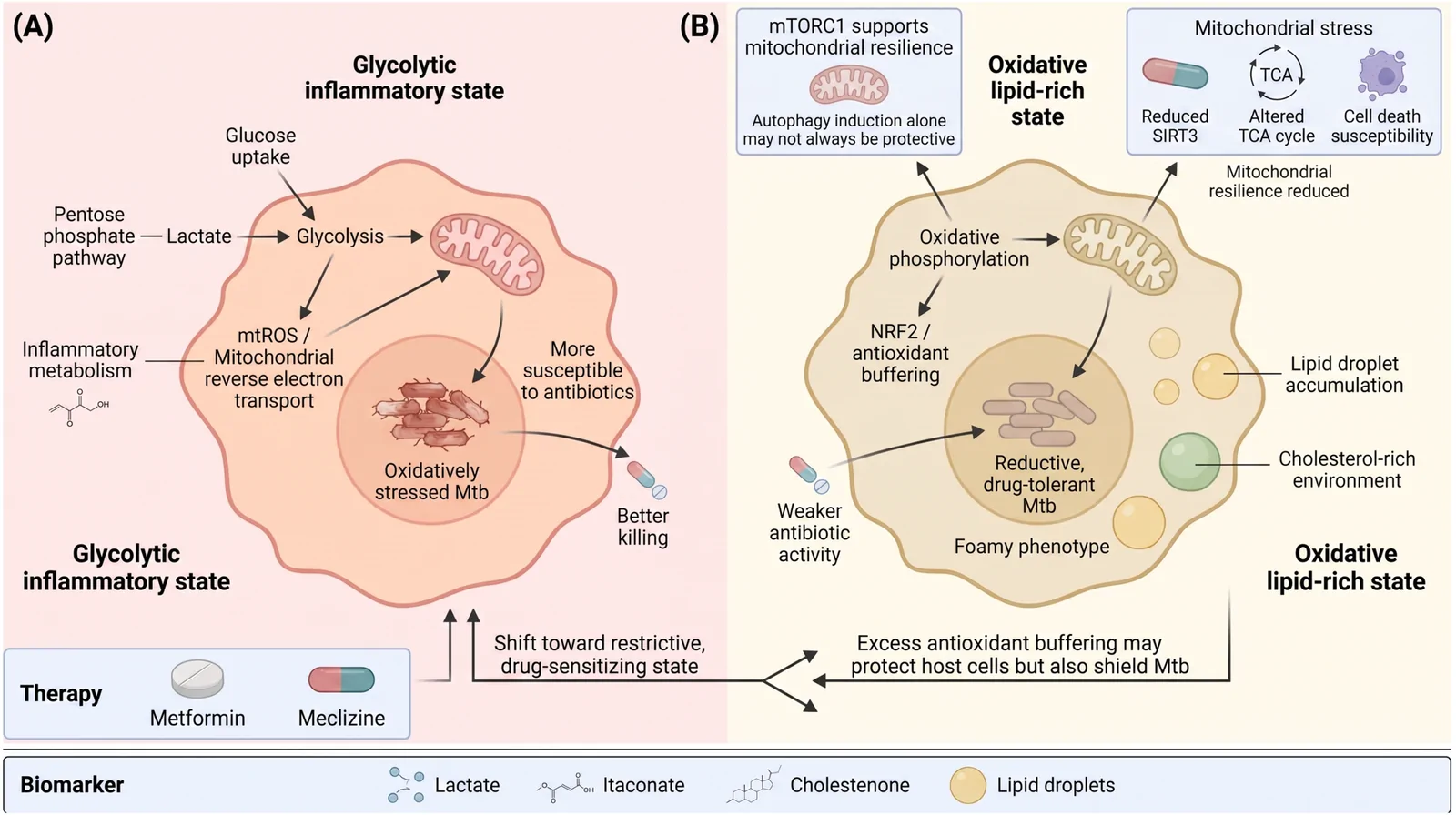
Macrophage metabolism influences bacterial redox state and antibiotic response, based on a 2025 preclinical study using intrabacterial redox reporters in mouse macrophages and mouse infection. **(A)** A glycolytic inflammatory state drives glucose uptake toward glycolysis, lactate production, and mitochondrial reverse electron transport, producing mtROS that oxidatively stresses Mtb and makes it more susceptible to antibiotics in this model; therapies like meclizine can promote this drug-sensitizing shift. **(B)** An oxidative lipid-rich state, characterized by high oxidative phosphorylation, lipid droplet accumulation, reduced SIRT3, and NRF2-mediated antioxidant buffering, provides a reductive environment that shields Mtb, leading to weaker antibiotic activity and drug tolerance in this model; therapies like metformin aim to reverse aspects of this state while maintaining mitochondrial resilience (e.g., via mTORC1). Candidate biomarkers such as lactate, itaconate, cholestenone, and lipid droplets help track these functional metabolic shifts. Whether this glycolytic-versus-oxidative dichotomy generalizes across macrophage populations, lesion contexts, and species, including humans, has not yet been established.

Meclizine shifted infected macrophages toward glycolysis, reduced bacterial redox heterogeneity, and decreased antibiotic tolerance in macrophage and mouse experiments without adverse interaction with first-line drugs (124). This is preclinical evidence. Metformin, discussed below, has human immunological, observational, randomized, and pharmacokinetic evidence and should not be presented as equivalent in mechanism or stage of development. The metabolic state of the host also changes antibiotic exposure at the bacterial level. Nutrient availability, redox buffering, and respiratory activity can alter whether intracellular Mtb is replicating, stressed, or tolerant without changing the nominal extracellular drug concentration. Foamy macrophages add a further complication because lipid stores can support bacterial carbon metabolism while changing host membrane trafficking and drug partitioning. These effects make metabolic HDT different from simple immune stimulation: the desired intervention must increase bacterial vulnerability while maintaining enough adenosine triphosphate (ATP), mitochondrial function, and antioxidant capacity to prevent destructive host-cell death. Paired metabolic flux, intrabacterial redox reporters, and antibiotic-killing measurements are therefore more informative than isolated host metabolite concentrations (115, 124). Antioxidant pathways therefore have two possible effects: they can limit host-cell injury but may also reduce oxidative pressure that improves killing of some bacterial populations. Metabolic interventions should be judged by macrophage viability, bacterial redox state and burden, lipid loading, and compatibility with the antibiotic regimen rather than by glycolysis or ROS alone.

Candidate blood or lesion biomarkers, including lactate, itaconate, cholestenone, and acyl-carnitines, remain exploratory until prospective clinical studies link them to treatment response or relapse (47, 112, 124). For treatment development, the aim is to shift macrophage metabolic states that permit persistence without sacrificing mitochondrial function or tissue integrity (Figures 6A,B).

## 9 Trained immunity, tolerance, and durable macrophage states

The question for trained immunity is whether a durable macrophage program improves early control without sustaining harmful inflammation. BCG exposure can induce nucleotide-binding oligomerization domain-containing protein 2 (NOD2)-dependent chromatin changes in circulating monocytes and reprogram hematopoietic stem and progenitor cells, extending innate memory beyond the lifespan of a single macrophage (125–127).

Innate memory can also arise locally after respiratory challenge, where alveolar macrophages may acquire durable glycolytic and antigen-presentation programs (128). Its effect depends on the disease setting: stronger cytokine responses may aid early containment yet worsen established inflammatory disease, while tolerance-like states can protect tissue but permit persistence (129–131). A recombinant BCG study reported mucosal and trained-immunity features but did not establish protection against subsequent Mtb challenge (132). For translation, durability is both an advantage and a safety concern. A short intervention that reprograms progenitors or resident macrophages could influence later cells without continuous dosing, but an inappropriate trained state could also prolong inflammatory injury. Route of exposure is likely to matter because intravenous, intradermal, and mucosal vaccination do not program the same cellular compartments (133). Studies of trained immunity in TB should therefore state which cell population is altered, how long the phenotype persists, whether the response is reproduced after differentiation, and whether subsequent Mtb challenge produces lower viable burden rather than only stronger cytokine release. These criteria are particularly important when trained-immunity concepts are used to support mucosal vaccination or long-acting HDT strategies.

Use of innate memory as therapy therefore requires a defined target population, timing window, and functional measurement that separates protective training from chronic inflammatory priming.

## 10 Granuloma ecology and lesion pharmacology

The lesion-level question is whether a therapy reaches the macrophage and bacterial populations that sustain disease. Granulomas vary in cellularity, vascularity, oxygen tension, pH, fibrosis, lipid content, and caseation (Figure 7A). These features determine macrophage state, bacterial location, and local drug exposure; a treatment effective in a uniform cell culture can therefore fail in a poorly penetrated lesion.

**Figure 7:**
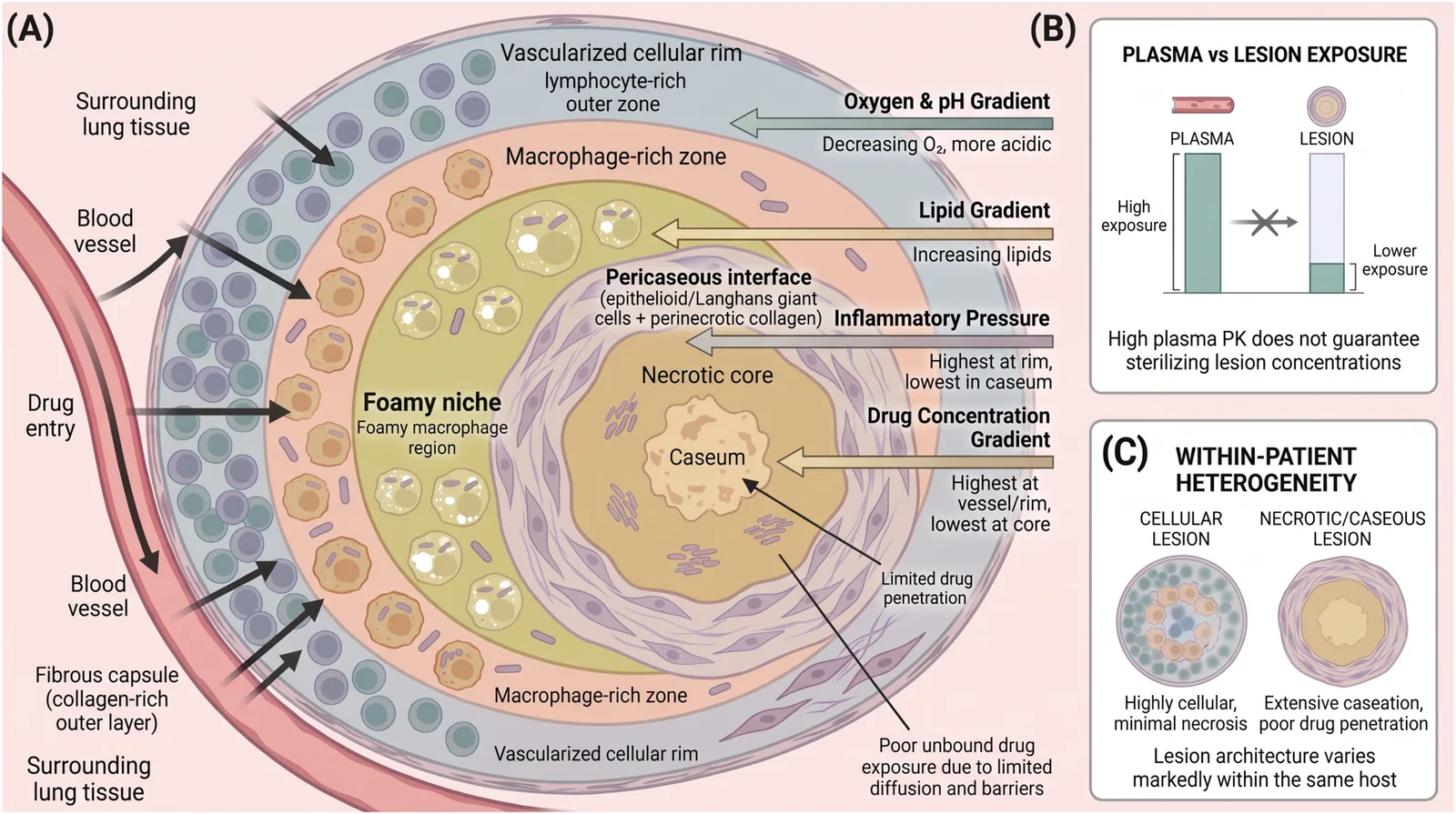
Granuloma architecture produces spatial gradients in host pressure and drug exposure. **(A)** A mature granuloma is concentrically organized. From the periphery inward, these zones comprise a collagen-rich fibrous capsule, a vascularized, lymphocyte-rich cellular rim, a macrophage-rich zone, a foamy macrophage niche, a pericaseous interface of epithelioid macrophages and Langhans giant cells with perinecrotic collagen deposition, and a central necrotic core containing caseum. This architecture creates gradients in which oxygen tension and pH decrease toward the center, while lipid content, diffusion barriers, and limitations on unbound drug availability increase. **(B)** Plasma versus lesion exposure highlights a major pharmacological challenge: high systemic plasma pharmacokinetics (PK) do not guarantee sterilizing drug concentrations at the lesion core due to limited penetration. **(C)** Within-patient heterogeneity illustrates that lesion architecture varies markedly, from highly cellular lesions with minimal necrosis to heavily necrotic and caseous lesions, impacting local drug efficacy and bacterial survival simultaneously within the same host.

Spatial pharmacology shows that plasma exposure does not reliably predict sterilizing activity. Mass-spectrometry imaging and lesion microdissection demonstrate different concentrations across cellular rims, necrotic regions, and caseum, and bacterial killing depends on adequate unbound drug at the site of persistent bacilli (Figure 7B) (31). Caseum is particularly restrictive because it contains lipids, proteins, necrotic debris, and extracellular bacilli in slow-growing or nonreplicating states. Drug binding and limited diffusion can produce low effective concentrations despite adequate plasma pharmacokinetics. Caseum-binding assays and computational predictions should therefore be calibrated against spatial measurements in animal or human lesions (32, 134, 135). Lesion heterogeneity also limits blood biomarkers. Spatial and single-cell studies show that macrophage composition and bacterial replication differ among granulomas within the same host (Figure 7C), so a blood inflammatory signature may not report the state of a poorly penetrated necrotic lesion (43–46).

Within-patient heterogeneity creates a further pharmacological problem: one regimen must act across several lesion types at the same time. A highly vascular cellular lesion may receive adequate drug while a caseous lesion in the same lung remains below the effective concentration. Host-directed changes can also alter this distribution by changing vascular permeability, fibrosis, lipid storage, or inflammatory cell recruitment. Consequently, plasma pharmacokinetics and whole-lung drug concentrations should not be used as substitutes for lesion exposure when a candidate is expected to act in caseum or poorly vascularized regions. Where tissue sampling is not feasible, imaging and model-based pharmacology should be anchored to lesion measurements obtained in appropriate animal or human studies (31, 134).

A lesion-based development approach should therefore combine primary human macrophages with granuloma or organoid systems, caseum-relevant assays, and models that reproduce necrosis. Pharmacokinetic studies should measure active drug in plasma, airway fluid, macrophages, cellular lesions, and caseum, and imaging should be linked to microbiological and functional outcomes (Figures 7A–C). These lesion differences also affect timing. As antibiotics reduce bacterial burden, the balance between containment and tissue repair changes. An HDT that is useful early may be harmful later, so regimen-specific and, where feasible, lesion-based dosing should be tested rather than assuming continuous adjunctive treatment is optimal.

## 11 Host-directed therapy: targeted modulation, not broad immune stimulation

The therapeutic question is which host abnormality should be corrected, in which patient, and at what stage of antibiotic treatment. HDT should therefore correct a defined host abnormality rather than stimulate immunity broadly. Candidate approaches affect autophagy, cytokines, eicosanoids, metabolism, redox control, cell death, or matrix remodeling, but their evidence levels and safety limits differ (Figure 8A) (24, 136–140). Statin evidence differs by agent and study design. Pravastatin’s mechanistic rationale is supported mainly by preclinical evidence: in human macrophages and mouse models it improved phagosomal maturation and enhanced first-line drug activity (141, 142). A different statin, atorvastatin, has since been tested in a randomized, open-label, multicenter phase 2b trial as an adjunct to standard-of-care in 150 treatment-naive patients with uncomplicated pulmonary tuberculosis (75 per arm). Adjunctive atorvastatin (40 mg daily for 2 months) was associated with a higher proportion of participants with negative sputum culture (97% vs. 85.1% with standard-of-care alone; *P* = 0.02) and a larger reduction in chest radiograph severity score (37% vs. 22%), with a comparable overall adverse-event profile aside from more frequent myalgia (16% vs. 5%) (143). This trial provides the strongest clinical signal to date for adjunctive statin therapy in TB, but it is a single, open-label study of one compound at one dose; given established differences in exposure, potency, metabolism, and drug interactions across statins, its findings should not be extrapolated to pravastatin or generalized to the statin class as a whole (141–143). Repurposed-drug screens directed at Mtb proteins are pathogen-directed rather than host-directed and should be evaluated separately (144).

**Figure 8:**
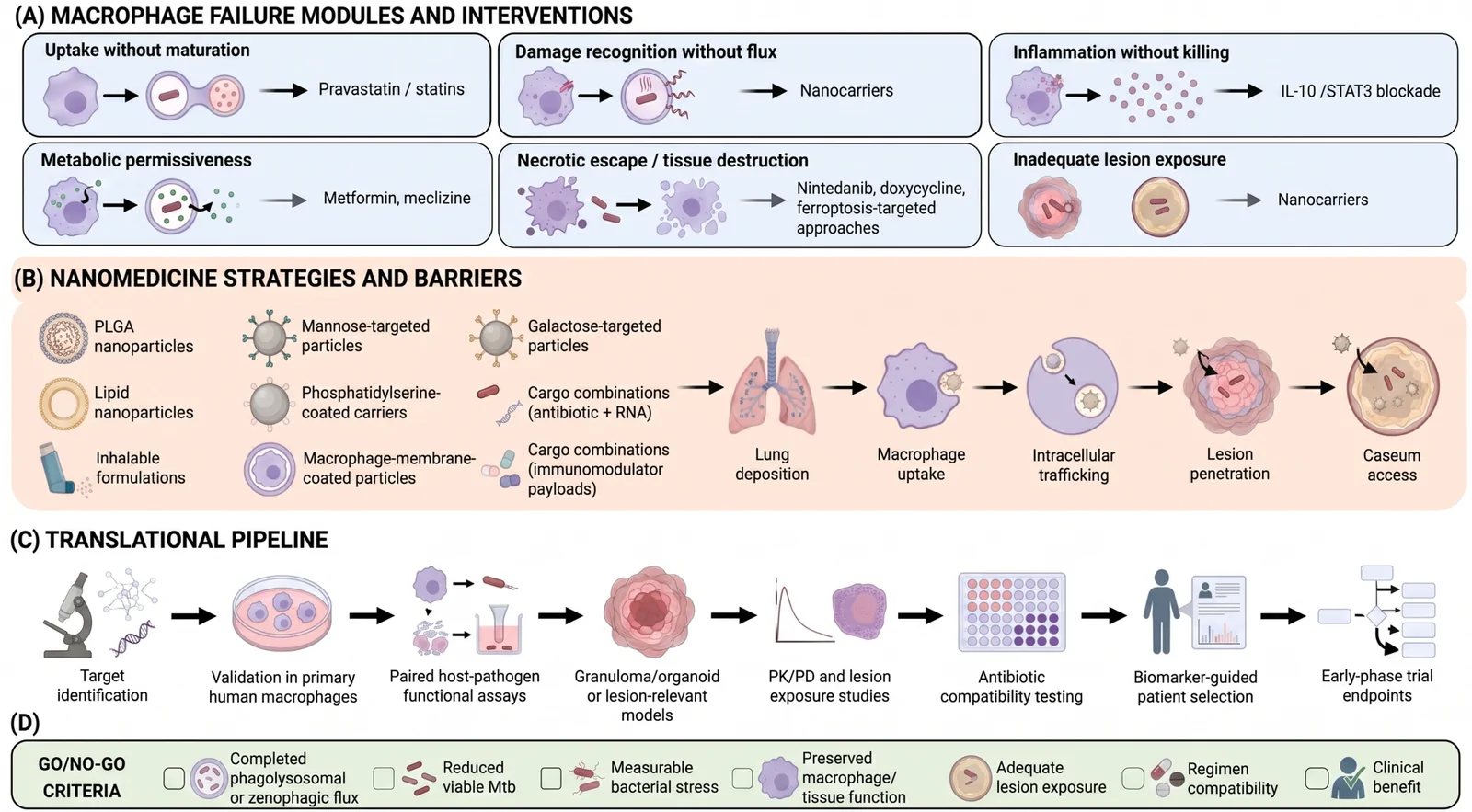
Matching macrophage defects to interventions and development tests. **(A)** Macrophage failure modules, such as uptake without maturation, damage recognition without flux, inflammation without killing, metabolic permissiveness, necrotic escape/tissue destruction, and inadequate lesion exposure, are paired with matched host-directed therapies (e.g., statins, IL-10/STAT3 blockade, metformin, nintedanib, doxycycline) or targeted nanocarriers. **(B)** Nanomedicine strategies (including PLGA, lipid, inhalable, and ligand-targeted particles) must traverse sequential barriers spanning lung deposition and macrophage uptake to intracellular trafficking, lesion penetration, and ultimate caseum access. **(C)** The translational pipeline requires progression from target identification and validation in primary human macrophages to paired host-pathogen functional assays, granuloma/organoid lesion-relevant models, PK/PD exposure studies, antibiotic compatibility testing, biomarker-guided patient selection, and rigorous early-phase trial endpoints. **(D)**, pre-specified go/no-go criteria demand proof of completed phagolysosomal/xenophagic flux, reduced viable Mtb, measurable bacterial stress, preserved macrophage/tissue function, adequate lesion exposure, regimen compatibility, and clearly defined clinical benefit.

Metformin has a broader evidence base than other candidates, especially in TB with diabetes, but the evidence differs importantly by study design and should not be treated as a single body of support. Mechanistic and human-immunology studies show that metformin alters phagocyte reactive-oxygen-species production and inflammatory signaling in both healthy subjects and TB-associated cohorts (145, 146). Separately, observational cohort and case studies associate metformin use with reduced mortality during TB treatment, a lower risk of incident TB among people with diabetes, and induced autophagy in diabetes-TB co-infection (147–149). In the randomized controlled trial that directly tested metformin’s clinical efficacy, however, adjunctive metformin did not improve the prespecified primary outcome of time to sputum culture conversion, although radiological and inflammatory signals were observed (33). A separate pharmacokinetic study shows that metformin can alter rifampicin, isoniazid, and pyrazinamide exposure, a regimen-compatibility consideration independent of efficacy (150). Taken together, the mechanistic and observational signals for metformin have not yet translated into a demonstrated benefit on the randomized trial’s primary microbiological endpoint; its strongest current rationale therefore remains specific to patient phenotype (particularly diabetes-associated TB) and drug regimen rather than a general antimycobacterial effect (33, 145–151). Clinical evidence for anti-inflammatory adjuncts is heterogeneous. Randomized studies and meta-analyses of vitamin D, phenylbutyrate, corticosteroids, pentoxifylline, N-acetylcysteine (NAC), cytokines, and related interventions do not support a common effect across the class (34, 35). A 2025 randomized trial (RIPENACTB) found that adjunctive NAC increased total glutathione and reduced lipid peroxidation and tumor necrosis factor-alpha without suppressing interleukin-10, and improved hemoglobin recovery and radiological and lung-function outcomes, but delayed time to negative sputum culture without an apparent adverse effect on overall treatment success; this dissociation between host-protective and bacterial-clearance measures illustrates why bacterial and host outcomes must be reported and interpreted separately rather than assumed to move together (152). A separate, small pilot study found that NAC supplementation significantly increased circulating interleukin-2 (IL-2) levels relative to controls in newly diagnosed pulmonary TB patients (*n* = 15 per arm), suggesting a possible immunomodulatory, T-cell-supportive effect; this finding is preliminary and exploratory and has not yet been linked to a bacteriological or clinical outcome (153).

The IL-10-signal transducer and activator of transcription 3 (STAT3) axis is supported by mechanistic preclinical studies. Aerosolized STAT3 or IL-10-receptor inhibitors reduced bacterial burden in chronically infected mice, and blockade of interleukin-10 receptor 1 (IL-10R1) improved pyrazinamide-mediated clearance (154, 155). These results justify further testing but do not establish safety or efficacy in patients, particularly where excess inflammation is already present. Nintedanib is another preclinical candidate. It showed direct anti-Mtb activity, increased macrophage autophagy through a Janus kinase 2 (JAK2)-STAT3-beclin 1 (BECN1) pathway, and improved chemotherapy outcomes and fibrosis in a chronic mouse model (156). Translation will require assessment of adverse effects, drug interactions, and whether antifibrotic activity weakens protective lesion structure.

Doxycycline provides randomized clinical evidence of a tissue-preserving biological effect: a short course reduced matrix metalloproteinase (MMP) activity, collagen and elastin destruction, and cavity-volume measures without a clear early reduction in mycobacterial load (11, 13). This supports matrix injury as a therapeutic target distinct from direct bacterial killing. Phase 2 data for CC-11050 and everolimus showed intervention-specific signals in lung-function recovery and biomarker responses, but no common efficacy pattern across the platform trial (10, 12). Conversely, myeloperoxidase (MPO) inhibition failed to improve bacterial burden or pathology in C3HeB/FeJ mice despite a mechanistic rationale from human neutrophils, showing how species-specific biology can invalidate a preclinical mechanism (157, 158).

Across these candidates, the strength of the claim should match the level of evidence. Mechanistic and preclinical findings justify target validation; human observational associations can define subgroups; randomized studies can test benefit; and clinical evidence of the intended biological effect should link target engagement to a prespecified microbiological, pathological, or functional endpoint. Table 2 summarizes these distinctions. Timing should be incorporated into this evaluation sequence. Early in treatment, an intervention that improves phagosome maturation, redox pressure, or bacterial stress may complement antibiotics. Later, when bacterial burden has fallen, the priority may shift toward limiting matrix destruction, fibrosis, or persistent inflammation. The same drug can therefore have different benefit-risk profiles at different stages. Trials should prespecify when the HDT is started and stopped and should test whether its pharmacodynamic effect overlaps with the period in which the targeted macrophage state is present. This is preferable to assuming that an adjunct should be given for the full duration of antimicrobial therapy.

**Table 2:** Translational assessment of selected host-directed therapy candidates.

Candidate	Target macrophage state	Evidence tier	Species/Model	Comparator	Primary endpoint	Biomarkers	Safety	Development stage	Refs.
Pravastatin/statins	Cholesterol handling, phagosome maturation, and autophagy	Preclinical (cell + *in vivo*) + randomized clinical	Human macrophages + mouse (pravastatin, preclinical); human, phase 2b RCT (atorvastatin)	Preclinical: untreated/vehicle control. RCT: standard-of-care alone (open-label, no placebo)	Preclinical: Rab7/LAMP1 colocalization, intracellular CFU. RCT (co-primary): safety (severe adverse events) and sputum culture negativity	Lipid signatures; CXR severity score (RCT secondary)	RCT: comparable overall adverse-event profile; more myalgia with atorvastatin (16% vs. 5%)	Preclinical (pravastatin); phase 2b completed (atorvastatin)	(141–143)
Metformin	AMPK/mTOR-linked metabolism, ROS, and phagocyte function	Mechanistic/observational + randomized clinical + PK	Human (mechanistic studies, observational cohorts, RCT)	RCT: standard-of-care alone	RCT: time to sputum culture conversion — not met	Glycemia; OCR/ECAR; ROS; inflammatory programs; CXR/inflammatory markers (RCT secondary, positive)	Not detailed in reviewed sources for the TB indication	Already-approved drug (T2DM); RCT completed for TB (primary endpoint negative)	(33, 145–151)
Vitamin D/phenylbutyrate and anti-inflammatory adjuncts	Cathelicidin/autophagy and inflammatory modulation	Randomized clinical (multiple trials) + meta-analysis	Human, multiple independent RCTs	Varies by trial (typically placebo or standard-of-care); not itemized per trial in this review	Heterogeneous across included trials; not itemized per trial in this review	25-hydroxyvitamin D; cathelicidin; CRP/cytokines; radiology	Not detailed in reviewed sources	Multiple RCTs completed; results heterogeneous/inconsistent across trials	(34, 35)
IL-10/STAT3 blockade	Reversal of immunosuppressive macrophage programs	Preclinical *in vivo*	Mouse (chronic TB infection; pyrazinamide-combination study)	Untreated/vehicle control	Bacterial burden (CFU) in mouse lung	STAT3 activity; NOS2; NADPH oxidase; lysozyme	Not yet tested clinically; hyperinflammation risk flagged mechanistically	Preclinical (mechanistic)	(154, 155)
Nintedanib	JAK2/STAT3/BECN1 autophagy and antifibrotic activity	Preclinical (cell + *in vivo*)	Macrophage (cell-based) + mouse (strain-dependent, chronic infection)	Untreated/vehicle control	Bacterial burden (CFU); lung pathology	Autophagic flux; fibrosis/MMP markers	Known hepatotoxicity risk (class effect)	Approved for other indications (idiopathic pulmonary fibrosis); preclinical for TB repurposing	(156)
Doxycycline	MMP-driven tissue destruction	Randomized clinical	Human, randomized phase 2	Not detailed in reviewed sources	Tissue-preserving effect (cavity volume/lung function); not primarily microbiological	Sputum/plasma MMPs; matrix fragments	Not detailed in reviewed sources	Phase 2 completed (positive tissue-effect signal); already an approved antibiotic being repurposed for host-directed effect	(11)
CC-11050/everolimus	PDE4- or mTOR-linked immune and repair programs	Randomized clinical (exploratory)	Human, randomized phase 2, exploratory platform trial	Platform-trial control arm; exact design not detailed in reviewed sources	Not detailed in reviewed sources; exploratory platform trial with multiple biomarker endpoints	FEV1; lung/blood biomarkers; microbiology	Small exploratory arms (underpowered for definitive safety conclusions)	Phase 2 (signal requiring confirmation)	(10, 12)
MPO inhibition	Neutrophil death-pathway modulation	Preclinical *in vivo* (negative)	Mouse	Untreated/vehicle control	Bacterial burden (CFU); pathology — no benefit observed	MPO activity; apoptosis/necrosis	Not detailed in reviewed sources; discontinued after negative efficacy result	Cautionary/discontinued despite mechanistic rationale; species-specific neutrophil biology limits human relevance	(157)

AMPK, AMP-activated protein kinase; BECN1, beclin 1; CFU, colony-forming unit; CRP, C-reactive protein; ECAR, extracellular acidification rate; FEV1, forced expiratory volume in 1 s; IL, interleukin; JAK2, Janus kinase 2; LAMP1, lysosome-associated membrane protein 1; MMP, matrix metalloproteinase; MPO, myeloperoxidase; mTOR, mechanistic target of rapamycin; NADPH, nicotinamide adenine dinucleotide phosphate; NOS2, inducible nitric oxide synthase; OCR, oxygen consumption rate; PDE4, phosphodiesterase 4; PK, pharmacokinetics; ROS, reactive oxygen species; STAT3, signal transducer and activator of transcription 3.

Before efficacy testing, each HDT program should specify the macrophage or lesion state to be corrected, the disease stage, partner regimen, exposure target, safety limit, and primary clinical objective. Few current candidates have demonstrated target engagement, lesion exposure, antibiotic compatibility, and patient benefit within one development program (159–165).

### 11.1 Nanotechnology-based targeting of macrophages and lesions

The delivery question is whether a formulation can cross the anatomical, cellular, and intracellular barriers that separate an administered drug from persistent Mtb (Figure 8B). Nanocarriers can improve stability, release, pulmonary deposition, or macrophage uptake, but each of these is an intermediate delivery property rather than proof of lesion or bacillary exposure (166, 167). Polymeric, lipid-based, and hybrid carriers have been developed for pulmonary or systemic delivery. Poly(lactic-co-glycolic acid) (PLGA) formulations are widely studied, including inhaled systems designed for alveolar macrophages. In preclinical studies, increased macrophage uptake or lower systemic exposure should be compared with an exposure-matched free-drug regimen and should not be assumed to mean delivery to the Mtb-containing compartment (168, 169). A nanocarrier’s contribution to therapy should also be classified by mechanism rather than described generically as “nanomedicine”: some carriers deliver a conventional antibiotic without modulating any host pathway (delivery-only platforms); some kill Mtb or infected cells through a physical mechanism such as heat or photodynamic reactive oxygen species rather than by correcting a host defect (physical-killing platforms); some modulate a macrophage pathway without an antibiotic payload (host-directed platforms); and a smaller number genuinely combine host-directed and antibiotic mechanisms within one carrier (true combination platforms). A targeting ligand or a favorable pharmacokinetic property does not by itself make a system host-directed, and Table 3 assigns each platform discussed below to one of these four categories.

**Table 3:** Translational comparison of macrophage- and lesion-targeted nanomedicine platforms.

Platform	Type	Category	Species/Model	Comparator	Primary outcome measured	Development stage	Main limitation	Refs.
PLGA-curcumin nanoparticles	Individual formulation	True combination (host-directed + antibiotic potentiation)	Mouse macrophage cell line (RAW264.7); rodent bioavailability studies	Untreated/free-drug control (standard preclinical design)	Autophagy, apoptosis, phagolysosomal fusion, and isoniazid potentiation	Preclinical	Cell-line dependence; limited lesion PK	(171)
Curdlan-PLGA nanoparticles	Individual formulation	Host-directed	Mouse macrophage cell line (RAW264.7); *in vitro* only, no *in vivo* model reported	Untreated infected macrophages; PLGA-only (unfunctionalized) nanoparticles; rifampicin (positive control)	TNF-alpha secretion (significantly increased, *P* < 0.0001); intracellular bacterial burden (luminescence/CFU) reduced but not statistically significant	Preclinical (*in vitro* only)	No *in vivo* validation yet; bacterial-burden reduction did not reach significance in this study	(172)
Phosphatidylserine-coated PCN-224-CpG particles	Individual formulation	Hybrid: host-directed (CpG/TLR9) + physical-killing (photodynamic)	*In vitro* (cell-based); species not specified in source	Untreated/vehicle control (standard preclinical design)	TLR9-biased immunity plus photodynamic ROS killing	Preclinical	Light delivery, tissue penetration, and off-target ROS	(173)
Macrophage membrane-coated AIE photothermal nanoparticles	Individual formulation	Physical-killing/imaging (not HDT)	Mouse (*in vivo* imaging/photothermal model)	Untreated/vehicle control (standard preclinical design)	NIR-IIb granuloma imaging and 1,064-nm photothermal killing	Preclinical	Thoracic light penetration and thermal control	(174)
Galactose-targeted circTRAPPC6B-rifampicin microparticles	Individual formulation	True combination (host-directed + antibiotic)	Macrophage (*in vitro*) + mouse (*in vivo*)	Untreated/free-rifampicin control (standard preclinical design)	Coordinates antibiotic exposure with autophagy, apoptosis, and M1-like polarization markers described in the source study	Preclinical	Nucleic-acid stability, immunogenicity, and manufacturing	(102, 103)
Inhalable PLGA systems	Platform class	Delivery-only	Formulation/delivery studies; species varies by source	Free-drug/exposure-matched control (standard preclinical design)	Local sustained delivery with lower systemic exposure	Preclinical	Aerodynamic performance, macrophage overload, and scale-up	(168)
Lipid nanocarriers	Platform class	Delivery-only	Multiple species; reviewed broadly, not platform-specific	Not applicable (review-level evidence)	Solubilize lipophilic/toxic drugs and prolong intracellular exposure	Preclinical	Caseum binding and incomplete intracellular trafficking	(170)
State-selective nanotherapy	Individual formulation	Host-directed	Macrophage (*in vitro*) + mouse (*in vivo*) TB models	Untreated/vehicle control (standard preclinical design)	Reduces KLK12/MMP signaling, pathology, and bacterial burden in the source model; does not establish a stable macrophage lineage	Preclinical	Marker stability, model generalizability, and off-target uptake	(175)
Engineered extracellular vesicles	Platform class	Delivery-only (unestablished)	Not TB-specific; platform reviewed generically	Not applicable (review-level evidence; TB efficacy not established)	Potential membrane-derived delivery of drugs or nucleic acids; TB-specific co-delivery efficacy is not established	Preclinical/early platform (pre-efficacy)	Donor variability, cargo heterogeneity, potency assays, and regulation	(167)
Inhaled mannose-targeted nanoemulsion	Individual formulation	True combination (antibiotic + host-directed apoptosis)	Mouse (aerosol performance + TB infection model)	Untreated/free-rifampicin control (standard preclinical design)	Combines local antibiotic exposure with host-directed apoptosis and granuloma targeting	Preclinical	Experimental infection model; repeat-dose safety and human lesion performance are unknown	(176)

AIE, aggregation-induced emission; circRNA, circular RNA; CpG, cytosine-phosphate-guanine; KLK12, kallikrein-related peptidase 12; MMP, matrix metalloproteinase; MOF, metal-organic framework; NIR-IIb, second near-infrared biological window; PK, pharmacokinetics; PLGA, poly(lactic-co-glycolic acid); ROS, reactive oxygen species; TLR9, Toll-like receptor 9.

Surface ligands such as mannose, phosphatidylserine, galactose, or beta-glucans can alter uptake, while size, charge, hydrophobicity, ligand density, protein corona, and material composition affect biodistribution. These variables must be linked to intracellular trafficking and lesion penetration rather than assessed only by total macrophage uptake (166, 167, 170). Most TB nanomedicine evidence remains preclinical, and the platforms below differ in whether they are host-directed, delivery-only, physical-killing, or true combination systems. Curcumin-loaded PLGA nanoparticles induce autophagy and phagosome-lysosome fusion while potentiating isoniazid activity, functioning as a true combination platform (host-directed cargo plus antibiotic potentiation) in cell-based and bioavailability studies (171). Curdlan-PLGA particles are a standalone host-directed platform, engaging Dectin-1 on RAW264.7 murine macrophages to significantly increase TNF-alpha secretion (*P* < 0.0001) without an antibiotic payload; intracellular bacterial burden was reduced but the change did not reach statistical significance, and this platform has so far only been tested *in vitro*, without an *in vivo* model (172). Phosphatidylserine-coated PCN-224 particles carrying cytosine-phosphate-guanine (CpG) oligodeoxynucleotides combine a genuinely host-directed element (CpG/Toll-like receptor 9 immune activation) with a physical-killing element (photodynamic reactive oxygen species generation), rather than acting through host-pathway correction alone (173). Macrophage-membrane-coated photothermal particles are a physical-killing and imaging platform: thermal ablation of Mtb-containing macrophages, not modulation of a host defense pathway, is the proposed mechanism, so this platform does not constitute host-directed therapy by itself (174). Evidence for these platforms comes from cell-based (curdlan-PLGA, PCN-224) or mouse *in vivo* (AIE photothermal) models, and each has specific safety and delivery constraints.

State-selective systems require especially careful interpretation. A nanoparticle carrying small interfering RNA (siRNA) against kallikrein-related peptidase 12 (KLK12) is a standalone host-directed platform, silencing a host inflammatory/matrix-remodeling gene without an antibiotic payload; it targeted macrophages described by the authors as M2-like and reduced MMP signaling, pathology, and bacterial burden in experimental TB (175). Here, M2-like is retained only as terminology from that model; it does not define a stable macrophage lineage in human TB. Galactose-targeted cellular microparticles carrying circTRAPPC6B and rifampicin are a true combination platform, coordinating a host-directed autophagy-inducing cargo with a co-delivered antibiotic, and reduced bacterial burden in macrophage and mouse models while altering autophagy and polarization markers (102, 103). These are preclinical findings; co-delivery may coordinate antibiotic and host-directed exposure, but it also adds requirements for RNA stability, cargo release, immunogenicity, batch consistency, and manufacturing control.

A 2026 study extended this concept to an inhaled mannose-targeted nano-emulsion co-delivering rifampicin with LCL161, a small-molecule inhibitor-of-apoptosis-protein antagonist intended to promote apoptotic clearance of infected macrophages; because it pairs an antibiotic with a host-directed apoptosis-promoting agent, this is a true combination platform rather than a delivery-only system. The formulation improved pulmonary and granuloma targeting and reduced bacterial burden and pathology in a mouse model (176). The study remains preclinical and used an experimental infection system, so clinical benefit, repeat-dose safety, and performance in heterogeneous human lesions are unresolved. By contrast, general lipid carriers and engineered extracellular vesicles are delivery-only platforms as currently used: lipid carriers can improve formulation of poorly soluble or toxic drugs and prolong intracellular exposure without modulating a host pathway, though lipophilic systems may also be retained in foamy macrophages or caseum, while engineered extracellular vesicles remain an early delivery platform with unresolved donor variability, cargo definition, potency assays, and regulation (167, 170).

Manufacturing and dosing constraints are part of the biological assessment. Inhaled particles must retain size distribution, drug loading, and release properties after nebulization, while systemically administered particles face protein-corona formation and hepatic or splenic clearance before reaching lung lesions. Ligand density can improve receptor-mediated uptake but can also change biodistribution or drive uptake by uninfected phagocytes. Repeated administration may alter innate responses to the carrier itself. For nucleic-acid cargo, potency also depends on intracellular release and molecular stability. These requirements explain why evidence of macrophage uptake in culture should be classified as early preclinical delivery evidence rather than as proof of lesion targeting or suitability for clinical use (168–170).

Complementary to delivery-platform development, several macrophage- and lesion-based screening efforts have identified new host-directed candidates independent of any nanocarrier. A chemical-genetics and machine-learning screen of macrophage infection models identified receptor tyrosine kinase inhibitors (dovitinib, AT9283, ENMD-2076) as host-directed antimicrobials acting on RTK signaling, independently validated by a kinome-wide siRNA screen (177). A screen of autophagy-modulating compounds in human macrophages identified the diphenylbutylpiperidine antipsychotics fluspirilene and pimozide as host-directed inhibitors of intracellular Mtb and Salmonella, acting through autophagy, TFEB-dependent lysosomal activation, and STAT5/CISH-mediated control of phagosomal acidification, with activity confirmed in MDR-Mtb strains and non-human-primate alveolar macrophages (178). The same group subsequently repurposed tamoxifen, identified through the same macrophage screening platform, as a host-directed inhibitor of Mtb and Salmonella acting via TFEB-dependent lysosomal activation rather than its canonical estrogen-receptor target, with efficacy confirmed in a zebrafish Mycobacterium marinum model (179). Most recently, a three-dimensional human tuberculoma bioplatform, designed to better approximate the granuloma microenvironment than conventional two-dimensional macrophage culture, screened host-directed and antibody-biosimilar libraries and identified six top-hit compounds (nitazoxanide, auranofin, doxycycline, dovitinib, 3’,4’-dichlorobenzamil, and AT9283) that reduced bacterial burden and resolved lesions across multiple mycobacterial strains; several hits, including dovitinib and AT9283, converged with the earlier two-dimensional RTK-inhibitor screen, and AT9283 was further validated in a mouse model (180). Together, these efforts illustrate a shift from single-target rationale toward unbiased, macrophage- and lesion-based discovery platforms for identifying host-directed candidates, complementing the nanocarrier delivery strategies discussed above.

At minimum, development studies should test storage and nebulization stability, batch reproducibility, protein-corona effects, uptake by primary human macrophage subsets, intracellular release, lesion penetration, compatibility with standard regimens, and repeat-dose toxicity. The comparator should be an exposure-matched free-drug regimen whenever feasible. Table 3 separates the biological rationale of current platforms from their evidence level and principal limitation. Carrier selection should be based on the barrier that must be overcome, not on macrophage uptake alone, and a system should advance only when active drug reaches the relevant lesion and intracellular compartment (Figures 8A–D).

## 12 Discussion: priorities for translation and future studies

Across the sections above, TB persistence emerges from a failure to coordinate uptake, membrane-damage sensing, cytokine and metabolic signaling, cell death, and antibiotic exposure within a defined macrophage state, rather than from failure of any single pathway (Table 1). Three practical priorities follow directly: pair every host-pathway measurement with a bacterial or functional readout in the same cells or lesions; build macrophage origin and lesion position into study design, since alveolar, interstitial, recruited, foamy, and repair-associated states are not interchangeable; and test HDTs and nanomedicines alongside the antibiotic regimens they are meant to accompany, with pharmacokinetic and lesion-exposure data included. Patient selection compounds these design choices: diabetes, HIV infection, nutritional status, lesion type, and antibiotic regimen can each shift the expected balance of benefit and harm, so biomarkers should demonstrate target engagement or identify a responsive subgroup rather than simply change during treatment.

Three specific controversies recur across this literature and deserve explicit synthesis rather than restatement within each mechanistic section. First, canonical and noncanonical LC3 responses are frequently conflated. Ca2+ leakage from damaged Mtb phagosomes can trigger V-ATPase- and ATG16L1-dependent LC3 lipidation on multiple membranes that limits membrane damage and bacterial replication without canonical delivery to lysosomes (73); because static LC3 puncta cannot distinguish this membrane-repair function from completed xenophagic degradation, a positive LC3 signal should be treated as evidence of membrane-stress control, not degradative flux, until flux and lysosomal delivery are directly measured (71, 73, 77). Second, cGAS-STING and TNF-alpha signaling are genuinely context-dependent rather than uniformly protective or pathological. ESX-1-dependent secretion of the Mtb effector EsxA drives both cGAS-STING-dependent type I interferon production and AIM2/NLRP3-inflammasome-dependent IL-1beta production, but through separable mechanisms: experimentally reducing EsxA secretion below the wild-type threshold selectively suppresses the type I interferon axis while leaving IL-1beta production intact, showing that these two ESX-1-triggered programs can be pharmacologically uncoupled rather than treated as a single readout of ESX-1 activity (68). Because type I interferon is associated with disease progression while IL-1beta and TNF-alpha are required for granuloma containment, the net effect of modulating this axis depends on which arm is engaged, at what infection stage, and in which macrophage population (24, 70–72, 104–106). Third, reactive oxygen and nitrogen species have a genuinely dual role that a single “antioxidant” or “pro-oxidant” framing cannot capture: ROS/RNS can directly stress intracellular Mtb and sensitize it to antibiotics, but the same oxidative burden can injure host tissue, and antioxidant therapy that protects tissue can simultaneously blunt the oxidative pressure that some antibiotic-killing mechanisms depend on (124). This tension is not only theoretical: a 2025 randomized NAC trial improved host-protective measures (glutathione, lipid peroxidation, TNF-alpha, hemoglobin recovery, radiological and lung-function outcomes) while delaying time to negative sputum culture, showing that host-protective and bacterial-clearance outcomes can move in opposite directions within the same patients and must be reported and interpreted separately rather than assumed to track together (152).

Recent translational and discovery-stage work illustrates how these principles are beginning to be applied. On the clinical side, a randomized phase 2b trial has now added human microbiological and radiological evidence for adjunctive atorvastatin, moving statin-based HDT beyond preclinical rationale for at least one compound (143), while the metformin and NAC trials discussed above show why randomized primary-outcome data must be distinguished from mechanistic and observational signal even for well-studied repurposed drugs (33, 152). On the discovery side, unbiased macrophage- and lesion-based screening platforms, spanning chemical-genetics and machine-learning screens of receptor tyrosine kinase inhibitors, a diphenylbutylpiperidine antipsychotic screen, tamoxifen repurposing, and a three-dimensional human tuberculoma bioplatform, have independently converged on overlapping hit compounds such as dovitinib and AT9283, suggesting these platforms are identifying reproducible host vulnerabilities rather than assay-specific artifacts (177–180). Extending these approaches to primary human cells, lesion-relevant three-dimensional systems, and paired host-bacterial readouts is now a more informative next step than further single-pathway target discovery in monoculture.

A practical early-phase trial can therefore use a four-stage endpoint plan. The first stage confirms exposure and target engagement; the second tests whether the predicted macrophage function changes; the third measures bacterial or lesion response; and the fourth assesses patient benefit and safety (Figure 8C). Failure at an early stage should prevent a favorable surrogate marker from being interpreted as therapeutic success (Figure 8D). Conversely, a tissue-preserving intervention should not be dismissed solely because it does not accelerate early sputum conversion if its prespecified objective is lung recovery. This separation is important for comparing metabolic HDT, antifibrotic treatment, and lesion-targeted delivery, which are intended to solve different problems (10–12). Combination design must also consider antagonism. Autophagy induction can be harmful if it weakens mitochondrial resilience; antioxidant therapy can protect tissue while reducing oxidative pressure that aids some antibiotic responses; and macrophage-targeting ligands can increase uptake by uninfected phagocytes. Sequential dosing, dosing directed to a defined macrophage state, or dosing based on lesion characteristics may therefore be preferable to simultaneous systemic activation of a host pathway.

Future studies should use primary human systems and lesion-relevant models, confirm target engagement and antibiotic compatibility, and connect host-state changes with local drug exposure and bacterial outcomes (15, 31, 124). This approach provides a clear basis for deciding which macrophage-directed strategies should progress from experimental mechanism to clinical evaluation.

## 13 Conclusions

Mtb persists in macrophage and lesion environments where microbial recognition, degradative trafficking, metabolic stress, control of cell death, adaptive immune pressure, and drug exposure are not always coordinated. A macrophage-state approach asks whether an intervention corrects a defined functional defect in the relevant lesion and antibiotic setting. Development should pair host and bacterial measurements, use primary human and spatially relevant models, verify local drug exposure, and separate microbiological, pathological, and functional clinical endpoints. Whether this strategy can shorten treatment, improve outcomes in drug-resistant or comorbid TB, or reduce post-TB disability should be tested as separate clinical hypotheses rather than inferred from pathway changes.
